# Transient and Persistent Metabolomic Changes in Plasma following Chronic Cigarette Smoke Exposure in a Mouse Model

**DOI:** 10.1371/journal.pone.0101855

**Published:** 2014-07-09

**Authors:** Charmion I. Cruickshank-Quinn, Spencer Mahaffey, Matthew J. Justice, Grant Hughes, Michael Armstrong, Russell P. Bowler, Richard Reisdorph, Irina Petrache, Nichole Reisdorph

**Affiliations:** 1 Integrated Department of Immunology, National Jewish Health and University of Colorado School of Medicine, Denver, Colorado, United States of America; 2 Department of Pharmacology, School of Medicine, University of Colorado Denver, Aurora, Colorado, United States of America; 3 Departments of Medicine and of Biochemistry and Molecular Biology, Indiana University, Indianapolis, Indiana, United States of America; 4 Department of Biostatistics and Informatics, University of Colorado Denver, Aurora, Colorado, United States of America; 5 Department of Medicine, National Jewish Health, Denver, Colorado, United States of America; 6 Department of Pediatrics, National Jewish Health, Denver, Colorado, United States of America; 7 Richard L. Roudebush VA Medical Center, Indianapolis, Indiana, United States of America; Institute of Lung Biology and Disease (iLBD), Helmholtz Zentrum München, Germany

## Abstract

Cigarette smoke exposure is linked to the development of a variety of chronic lung and systemic diseases in susceptible individuals. Metabolomics approaches may aid in defining disease phenotypes, may help predict responses to treatment, and could identify biomarkers of risk for developing disease. Using a mouse model of chronic cigarette smoke exposure sufficient to cause mild emphysema, we investigated whether cigarette smoke induces distinct metabolic profiles and determined their persistence following smoking cessation. Metabolites were extracted from plasma and fractionated based on chemical class using liquid-liquid and solid-phase extraction prior to performing liquid chromatography mass spectrometry-based metabolomics. Metabolites were evaluated for statistically significant differences among group means (*p*-value≤0.05) and fold change ≥1.5). Cigarette smoke exposure was associated with significant differences in amino acid, purine, lipid, fatty acid, and steroid metabolite levels compared to air exposed animals. Whereas 60% of the metabolite changes were reversible, 40% of metabolites remained persistently altered even following 2 months of smoking cessation, including nicotine metabolites. Validation of metabolite species and translation of these findings to human plasma metabolite signatures induced by cigarette smoking may lead to the discovery of biomarkers or pathogenic pathways of smoking-induced disease.

## Introduction

A biological marker (biomarker) is a characteristic that is objectively measured and evaluated as an indicator of normal or pathogenic biological processes, or of pharmacologic responses to a therapeutic intervention [1]. Biomarkers identified in biological fluids such as plasma can be used for early disease diagnoses or to identify those at risk for disease development, which could lead to behavior modification or clinical interventions that may impact disease prognosis. Metabolomics has become a critical tool in clinical research for identifying disease biomarkers associated, for example, with heart disease [2], with cancers of the prostate [3] or lung [4], and with nonalcoholic fatty liver disease [5].

The respiratory tract is the first target of cigarette smoke inhalation and cigarette smoking is a major risk factor for diseases of the airways such as chronic obstructive pulmonary disease (COPD) [6] and head, neck, and lung cancers [7]. It is well established that cigarette smoking is also a risk factor for many systemic diseases, including cardiovascular disease and numerous cancers (e.g. bladder or pancreas), suggesting significant pathogenicity of circulating toxic components or metabolites of absorbed cigarette smoke. However, not all smokers develop disease and progression of diseases such as COPD may persist despite smoking cessation. These observations suggest a critical need for determining pathways through which smoking can lead to disease and for the discovery of useful biomarkers of disease phenotype, severity, and progression.

There are currently few published studies exploring metabolome changes due to smoking. Rather, much of the literature has focused on developing methods to identify compounds in cigarette smoke using techniques such as liquid chromatography tandem mass spectrometry [8], [9] and gas chromatography mass spectrometry [10]. Other investigators have looked at specific cigarette smoke metabolites in biological fluids as biomarkers of cigarette smoke exposure [11]–[13], while other studies have focused on understanding enzyme activities [14] or nicotine biosynthesis [15] of tobacco plants. Xu et al [16] studied the effects of smoking on the human serum metabolome using a targeted metabolomics approach of 140 metabolites with a focus on amino acids and phosphotidylcholines (PC). To our knowledge, no study has yet examined how cigarette smoke affects the entire plasma metabolome. Using state of the art untargeted metabolomics as previously described by Yang et al [17] applied to a cigarette smoking model in mice, we sought to examine changes in the plasma metabolome that are induced by chronic cigarette smoking, and to determine if these changes are stable or transient following smoking cessation.

## Materials and Methods

### 1 Animal studies

#### Ethics Statement

Animal studies were approved by the Animal Care and Use Committee of Indiana University.

DBA/2J mice (Jackson Labs; male; age 12–14 weeks; n = 2–3 per group) were exposed to ambient air or cigarette smoke as previously described by Schweitzer et al [18]. These mice have been shown to be susceptible to cigarette smoke-induced emphysema-like disease following at least 4 months of exposure [18], [19], also demonstrated in this mouse cohort by increased mean linear intercepts (Figure S1). Briefly, mice were exposed 5 hours per day, 5 days a week to 11% mainstream and 89% side-stream smoke from reference cigarettes (3R4F; Tobacco Research Institute, Kentucky) using a Teague 10E whole body exposure apparatus (Teague Enterprise, CA) with monitored suspended particulates (average 90 mg/m^3^) and carbon monoxide (average 350 ppm). Five groups of mice were analyzed: 1) ambient air-only exposed at four or six months (controls, n = 3), 2) cigarette smoke-exposed for 4 months (CS-4mo, n = 2), 3) cigarette smoke-exposed for 6 months (CS-6mo, n = 3), and 4) cigarette smoke-exposed for 4 months followed by 2 months in ambient air (CS-cessation, n = 3). At the end of experiments, the mice were euthanized and the plasma was collected by cardiac puncture and then snap-frozen in liquid nitrogen and stored at −80°C.

### 2 Chemicals and standards

Solvents used for metabolite extraction and LC/MS analysis were of HPLC or LC/MS-grade. Isopropyl alcohol and water were purchased from Honeywell Burdick & Jackson (Muskegon, Michigan). Methyl tert-butyl ether was purchased from J.T. Baker (Central City, Pennsylvania). Acetonitrile, methanol, chloroform, hexane, and acetic acid were purchased from Fisher Scientific (Fair Lawn, New Jersey).

The neutral and phospholipid standards – Triglyceride d5-(20∶0/20∶1(11Z)/20∶0)), C17 ceramide (d18∶1/17∶0), 1,2-dipentadecanoyl-*sn*-glycero-3-phosphocholine (15∶0 PC), and 1,2-diheptadecanoyl-*sn*-glycero-3-phosphoethanolamine (17∶0 PE) – were purchased from Avanti Polar lipids Inc. (Alabaster, Alabama). The aqueous standard – creatinine-d3 – was purchased from Sigma Aldrich (St. Louis, MO).

Solid phase extraction (SPE) 12-position vacuum manifold and Strata NH_2_ (55 µM, 70 Å) 100 mg/mL SPE cartridges were purchased from Phenomenex (Torrance, California). Glass pipette tips, plastic pipette tips and microcentrifuge tubes were purchased from Fisher Scientific (Fair Lawn, New Jersey). Pyrex glass culture tubes were purchased from Corning Incorporated (Corning, New York).

### 3 Sample Preparation/Extraction

Standards stored at −20°C and samples stored at −80°C were thawed and kept on ice prior to sample preparation. Samples (n = 2–3/group) were prepared using the liquid-liquid extraction (LLE) and solid-phase extraction (SPE) method previously described by Yang et al [17] with the following changes: subsequent to the extraction steps, the neutral lipid fraction was resuspended in 400 µL 1∶1 chloroform: methanol, the phospholipid fraction was resuspended in 400 µL methanol, and the aqueous fraction was resuspended in 100 µL 3% acetonitrile with 0.1% formic acid. Blank samples and a pooled plasma sample were prepared with each batch for sample preparation quality control (QC) purposes. A single pooled plasma sample was prepared at the beginning of the study for use as an instrument QC sample.

### 4 Liquid chromatography/mass spectrometry

The aqueous fraction samples were analyzed in positive ESI mode at a flow rate of 0.3 mL/min with scan range 50–1050 m/z and scan rate 2.01. 10 µL of sample was injected onto a Zorbax SB-AQ analytical column (1.8 micron, 2.1×50 mm) with a narrow bore SB-AQ guard cartridge (5 micron, 2.1×12.5 mm, 80 Å). Mobile phase A was water with 0.1% formic acid and mobile phase B was 90∶10 acetonitrile: water with 0.1% formic acid. Gradient elution was as follows: 0–3 minutes, 2% B, 3–5 minutes 2–40% B, 5–20 minutes 40–100% B, 20–30 minutes 100% B, followed by column re-equilibration. MS source conditions were as follows: gas temperature 300°C, gas flow 10.0 L/min, nebulizer pressure 45 psi, fragmentor 160 V, ESI capillary voltage 4000 V with reference masses 121.05087 and 922.00979 (Agilent reference mix).

The neutral lipid and phospholipid fractions were analyzed in positive ESI mode at a flow rate of 0.5 mL/min with scan range 60–1600 m/z and scan rate 2.01. 10 µL of sample was injected onto an SB-C3 analytical column (5 µM, 2.1×50 mm) with a narrow bore SB-C3 guard cartridge (5 micron, 2.1×1.5 mm, 300 Å). Mobile phase A was water with 5 mM ammonium acetate and mobile phase B was isopropyl alcohol with 5 mM ammonium acetate. Gradient elution was as follows: 0–2 minutes, 30% B, 2–11 minutes 30–100% B, 11–15 minutes 100% B, followed by column re-equilibration. MS source conditions were as follows: gas temperature 300°C, gas flow 7.0 L/min, nebulizer pressure (neutral fraction: 40 psi, phospholipid fraction: 50 psi), fragmentor (neutral fraction: 120 V, phospholipid fraction: 140 V), ESI capillary voltage 2000 V with reference masses 121.05087 and 922.00979 (Agilent reference mix).

Sample analysis was performed on a Time-of-Flight mass spectrometer (6210 TOF-MS, Agilent Technologies) with electrospray source coupled to a high performance liquid chromatography system (Agilent 1200 Series) as described previously [17]. All samples were randomized and injected in triplicate. The analytical quality of the sample preparation, method and instrument were evaluated by injecting the sample prep and instrument QC samples in triplicate during each batch of samples analyzed using TOF, for a total of 7 batches.

The quality control internal standards described in methods section 2 were analyzed to ensure mass accuracy within 3 ppm, retention time reproducibility of less than +/−5% and HPLC pressure curve reproducibility of less than +/−5% at 10 time points. Sample batches that did not meet these criteria were re-analyzed.

### 5 Data and statistical analysis

Prior to quantitative analysis, data were evaluated for sample preparation reliability and retention time reproducibility by examining the spiked internal standards described in methods sections 3 and 4.

Data was extracted using the following parameters in Mass Hunter software (Agilent Technologies): Find by Molecular Feature algorithm, single charge, proton and sodium adducts only in positive mode. Data were imported into Mass Profiler Professional software (MPP, Agilent Technologies) for mass (15 ppm) and retention time alignment (0.2 minutes), and data filtered by selecting features which were present in 2 out of 3 technical replicates. Data from sample preparation blanks and instrument blanks were background subtracted to eliminate noise from contaminants. This resulted in 7589 molecular features.

Because LCMS data can result in missing values [20], data was further processed using the ‘Find by Formula’ algorithm parameters (+H and +Na adducts for positive ionization mode, −H for negative ionization mode, charge states limited to 1, and absolute height >3000 counts). The final data set was then re-imported into MPP for analysis, including normalization (‘75% percentile shift’ normalization algorithm). The ‘Find by Formula’ algorithm combined multiple features such as adducts and dimers into a single compound which resulted in 3045 total compounds.

Statistical analysis was performed on the 3045 compounds (1561 in the aqueous fraction, 886 in the neutral fraction and 598 in the phospholipid fraction). ANOVA using Student-Newman-Keuls (SNK) post-hoc test or Student’s unpaired t-test were applied. Benjamini-Hochberg false discovery rate (FDR) multiple testing correction using asymptotic p-value computation with a corrected p-value cut-off of 0.05 was used. The list of 3045 compounds was reduced to 656 after FDR<0.05. Fold change cut-off of 1.5 was then applied to this list resulting in 584 compounds following fold change analysis. This final list of significant metabolites were exported into Mass Hunter Quantitative Analysis Software (Agilent) to further reduce the list of potential false positives by removing retention time outliers using a 0.30 minute cut-off window. This final list of 222 metabolites was then re-imported into MPP for annotation, data summary, and visualization purposes.

Finally, annotated compounds and their normalized abundance values were exported to Excel Professional Plus 2010 (Microsoft Corporation, Redmond, WA) for graphing.

### 6 Identification of plasma metabolites

ID Browser within the Mass Profiler Professional software was used to tentatively identify metabolites (Table S1). This software utilizes an in-house database comprising data from Metlin, Human Metabolome Database (HMDB), Kyoto Encyclopedia of Genes and Genomes (KEGG) and Lipid maps and applies isotope ratios, accurate mass, chemical formulas, and scores to provide preliminary identifications (Table S2). Chosen elements for molecular formula generation were: C, H, N, O, S, and P. A 10 ppm mass error cutoff was used with a neutral mass range up to 2000 Da and positive ions selected as H, Na, K, and NH_4_. The database identifications were limited to the top 10 best matches based on score, and charge state was limited to a maximum of 2. Selective solvent extraction implemented during the sample preparation step was applied for additional metabolite information based on lipid chemical class. All identifications are Metabolomics Standards Initiative (MSI) level 2 based on the proposed minimum reporting by Sumner et al [21].

## Results and Discussion

### 1 Quality Control

Quality control (QC) samples were analyzed (n = 31 in the aqueous fraction, n = 31 in the neutral lipid fraction, and n = 32 in the phospholipid fraction) and spike-in standards of known concentration were used to test the LLE and SPE fractionation method efficiency. As expected, only the aqueous spike (creatinine-d3) was detected in the aqueous fraction. Neutral spike-in compounds (TG-d5 and ceramide C17) were detected in the neutral lipid fraction, although the aqueous spike (creatinine-d3) and a phospholipid spike (15∶0 PC) were also detected in this fraction, in much lower abundance (29-fold and 2.3-fold lower, respectively). Similarly, the phospholipid spike-in compounds (15∶0 PC and 17∶0 PE) were detected in the phospholipid fraction, along with small amounts of the aqueous standard creatinine-d3 at 2.5 times lower abundance than in its aqueous fraction. Additionally, the neutral lipid standard ceramide C17 was detected in the phospholipid fraction at 2960-times lower abundance compared to the neutral fraction. The contamination noted was most likely due to minor carryover on the SPE column during the lipid fractionation process. Table 1 shows the reproducibility of the spike-in standards for each QC fraction. These standards were used for quality control purposes rather than for normalization. Although minimal, the few endogenous metabolites which were present in multiple fractions in the sample data were discarded for final quantitative analysis.

**Table 1:** Quality Control using spiked-in standards for each fraction.

Fraction	Standard	n	Average RT (min)	% CV (RT)	Average Area	% CV (area)
Aqueous	Creatinine-d3	31	0.724	0.80%	2217311	3.83%
Neutral lipid	Triglyceride-d5	31	11.299	0.07%	4837032	9.92%
	Ceramide C17	31	7.425	0.19%	12736707	7.86%
Phospholipid	15∶0 PC	32	7.013	0.63%	1248929	9.32%
	17∶0 PE	32	7.271	0.35%	517234	7.99%

Retention time % CVs are less than 1% and peak area % CVs are less than 10%. Aqueous fraction was analyzed using SB-C3 analytical column and neutral lipid and phospholipid fractions were analyzed using a C18 analytical column on an Agilent 6210 ESI-TOF. Data was analyzed using Agilent Mass Hunter Qualitative and Quantitative Analysis software.

### 2 Mouse plasma metabolomics

#### 2.1 Nicotine metabolites in mouse plasma

**Figure 1:**
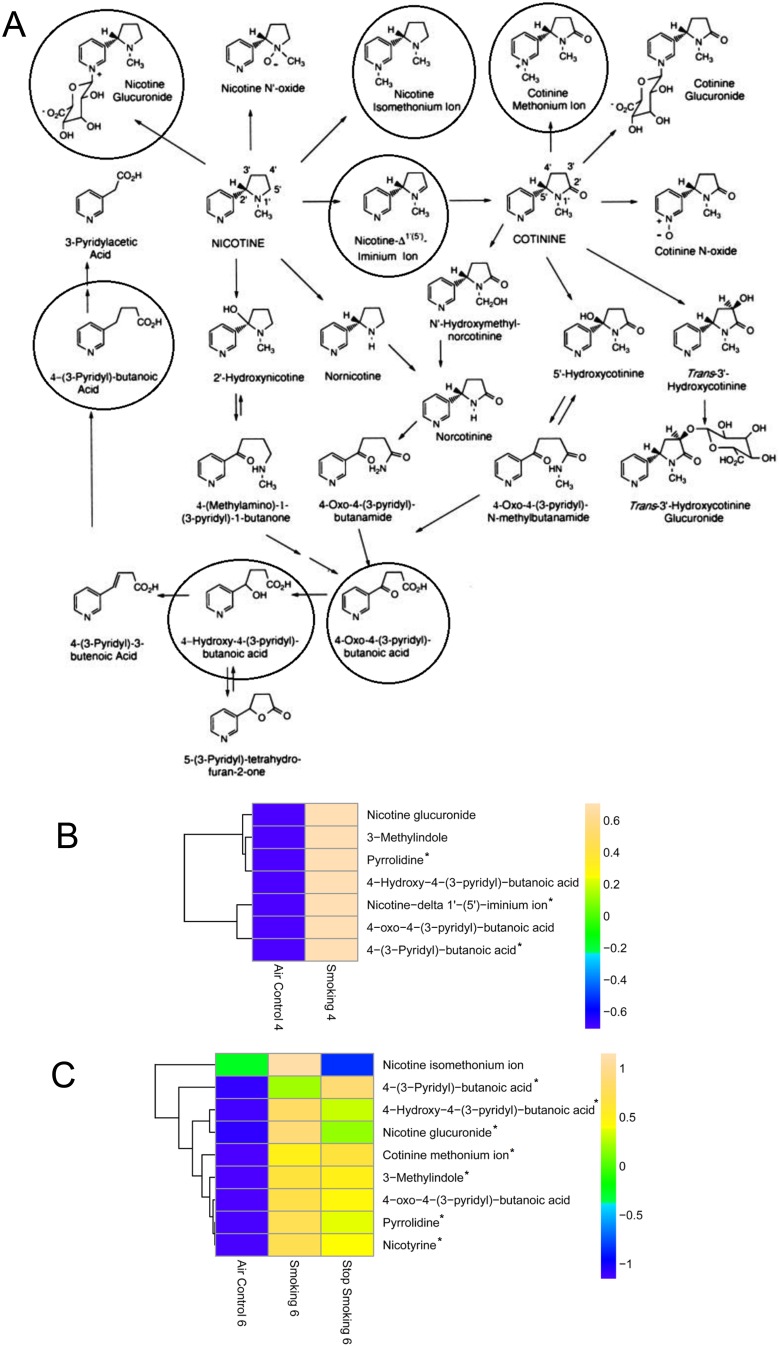
(A) Nicotine pathway and heat maps of CS metabolites in (B) four month and (C) six month comparisons. CS = cigarette smoke. Pathway diagram reprinted with permission from Hukkanen et al. Circled compounds represent the metabolites identified in the plasma of mouse samples in the current study. For each heat map, blue represents a decrease in mean metabolite abundance, and yellow/beige represents an increase in mean metabolite abundance. For example, nicotine glucuronide is increased in smoking in both the 4- and 6- month smoking mice. Samples were analyzed on an Agilent 6410 ESI-TOF in positive ionization mode, data was processed using Mass Profiler Professional, and quantitative data was obtained using Mass Hunter Quantitative analysis software. Tentative identification was performed using ID Browser within the Mass Profiler Professional software. ID Browser in-house database is comprised of Metlin, Lipid Maps and HMDB. * indicates *p*-value≤0.05 between the air control and cigarette smoking groups. Differences between other nicotine pathway metabolites are also shown but did not reach statistical significance.

We first investigated whether known cigarette smoke components, such as nicotine metabolites, could be detected as indicators of cigarette smoke exposure and to evaluate their persistence in plasma 2 months following cessation of smoking. Water soluble cigarette smoke metabolites were detected in the aqueous fraction of mouse plasma as shown in Figure 1A, adapted from Janne Hukkanen, Peyton Jacob III, and Neal L. Benowitz, Metabolism and Disposition Kinetics of Nicotine, Pharmacol Rev March 2005 57∶79–115.

Nicotine itself was not detected in any of the plasma samples. This is likely due to its short half-life in plasma (2–3 hours) compared to urine (11 hours) [22] and the fact that plasma collection was performed 16–24 h following active cigarette smoke exposure. Nicotine metabolites with similarly short half-lives such as anabasine, anatabine, nornicotine, and nicotine isomethonium ion were undetectable or showed minimal elevations in plasma of mice exposed to cigarette smoke. In contrast, the levels of nicotine glucuronide paralleled smoking exposure history and were highly elevated in CS-6mo samples compared to AC-6mo samples (*p* = 0.00043), but were also elevated in CS-cessation compared to AC-6mo (*p* = 0.027). Although levels declined in the CS-cessation group compared to the CS-6mo group, this was not significant (*p* = 0.083). Interestingly, nicotine iminium ion was present in CS-4mo but was absent in the CS-6mo plasma, suggesting that with continued smoking, other routes in the nicotine metabolism pathway, such as conversion to nornicotine, may become more predominant. Nicotyrine also exhibited levels which paralleled smoking exposure with an increase in CS-6mo and CS-cessation compared to AC-6mo (p = 0.013 and p = 0.0037), with a possible decrease in the CS-cessation compared to CS-6mo group (p = 0.57). Additionally, cotinine methonium ion was undetected in AC-4mo and CS-4mo samples, but was present in CS-6mo and CS-cessation indicating that cotinine methonium ion accumulates after chronic cigarette smoke exposure and can still be detected for at least two months following smoking cessation.

Other nicotine metabolites measured were 4-oxo-4-(3-pyridyl)-butanoic acid, which was absent in half of the air controls but present in the smoking samples and 4-hydroxy-4-(3-pyridyl)-butanoic acid, which was detected in low amounts in AC-4mo but was variably elevated in the CS-4mo samples (not reaching statistical significance) and significantly elevated in CS-6mo and CS-cessation plasma (p = 0.0027 and p = 0.00069 respectively, compared to AC-6mo). Similarly, the 4-(3-pyridyl)-butanoic acid metabolite was elevated following prolonged in CS-6mo and CS-cessation samples (p = 0.0017 and p = 0.0012 respectively, compared to AC-6mo). These metabolites are relevant to human CS exposure, since 4-oxo-4-(3-pyridyl)-butanoic acid has been previously reported in human urine at an average concentration of 228 ng/mL [23] and both the (S) and (R) enantiomers of 4-hydroxy-4-(3-pyridyl)-butanoic acid have been reported in human urine at average concentrations of 14.1 ng/ml and 1120 ng/ml respectively [23].

In addition to nicotine metabolites, additional exogenous compounds representing other tobacco smoke metabolites were identified in mice chronically exposed to cigarette smoke (Figure 1). The identification of these compounds may inform our understanding of tobacco smoke metabolite clearance and persistence. Pyrrolidine, found naturally in the leaves of tobacco and present in cigarette smoke was significantly elevated in the CS-4mo (p = 0.000025 vs. AC-4mo) and in the CS-6mo group (p = 0.022 vs. AC-6mo). After smoking cessation, pyrrolidine levels declined in plasma compared to those in mice that continued smoking (the CS-6mo group), however this difference was not statistically significant (p = 0.62) suggesting a slower clearance from plasma than other metabolites. The low levels of pyrrolidine detected in the plasma of AC-4mo group may be due to its use as a flavoring ingredient, which is present in small quantities in a range of food products including animal food [24]. We also detected 3-methylindole, another component of cigarette smoke [25], [26] being elevated in the CS-6mo and CS-cessation groups compared to AC-6mo (p = 0.0044 and p = 0.0073 respectively), with a non-significant decrease in CS-cessation compared to CS-6mo (p = 0.77).

To our knowledge, other than nicotyrine, 4-hydroxy-4-(-pyridyl)-butanoic acid, and 4-oxo-4-(3-pyridyl)-butanoic acid, biological fluid concentrations of the other seven of the cigarette smoke metabolites detected with our methodology (Figure 1) have not yet been reported in the literature. Overall, our data indicate that the majority of cigarette smoke metabolites detected in the plasma of mice exposed to smoking decreased after 2 months following cessation with the exception of 4-(3-pyridyl)-butanoic acid and cotinine methonium ion. In addition to providing insight into the systemic absorption of cigarette smoke components and their long term metabolism, the data presented support the reliability of our methodology used to characterize plasma metabolome changes in response to cigarette smoking.

#### 2.2 Impact of cigarette smoking on endogenous plasma metabolites

**Figure 2:**
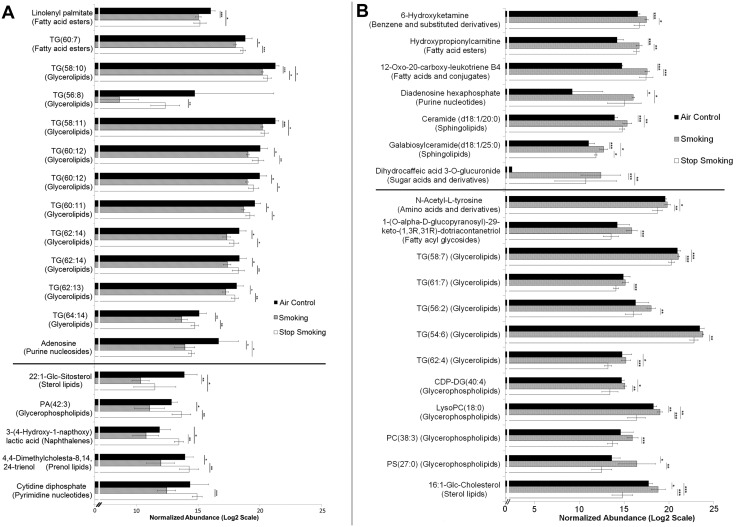
Metabolites which were (A) reversibly decreased and (B) reversibly increased with smoking. In panel A (top), differentially regulated metabolites were higher in air controls, decreased with smoking, and increased toward air control levels following smoking cessation. In panel A (bottom), differentially regulated metabolites were higher in air controls, decreased with smoking, and surpassed air control levels following smoking cessation. In panel B (top) differentially regulated metabolites were lower in air controls, increased with smoking, and decreased following smoking cessation. In panel B (bottom), differentially regulated metabolites were lower in air controls, increased with smoking, and decreased beyond air control levels following smoking cessation. Samples and data were analyzed as described in the methods section. TG = triglyceride, CDP = cytidine-diphosphate, DG = diglyceride, PC = phosphatidylcholine, PS = phosphatidylserine, TG = triglyceride, PA = phosphatidic acid. Fold change ≥1.5; *p*-value≤0.05, x-axis = Log2 normalized abundance scale; Error bars represent 95% confidence; Significance values *≤0.05, **≤0.01, ***≤0.001.

**Figure 3:**
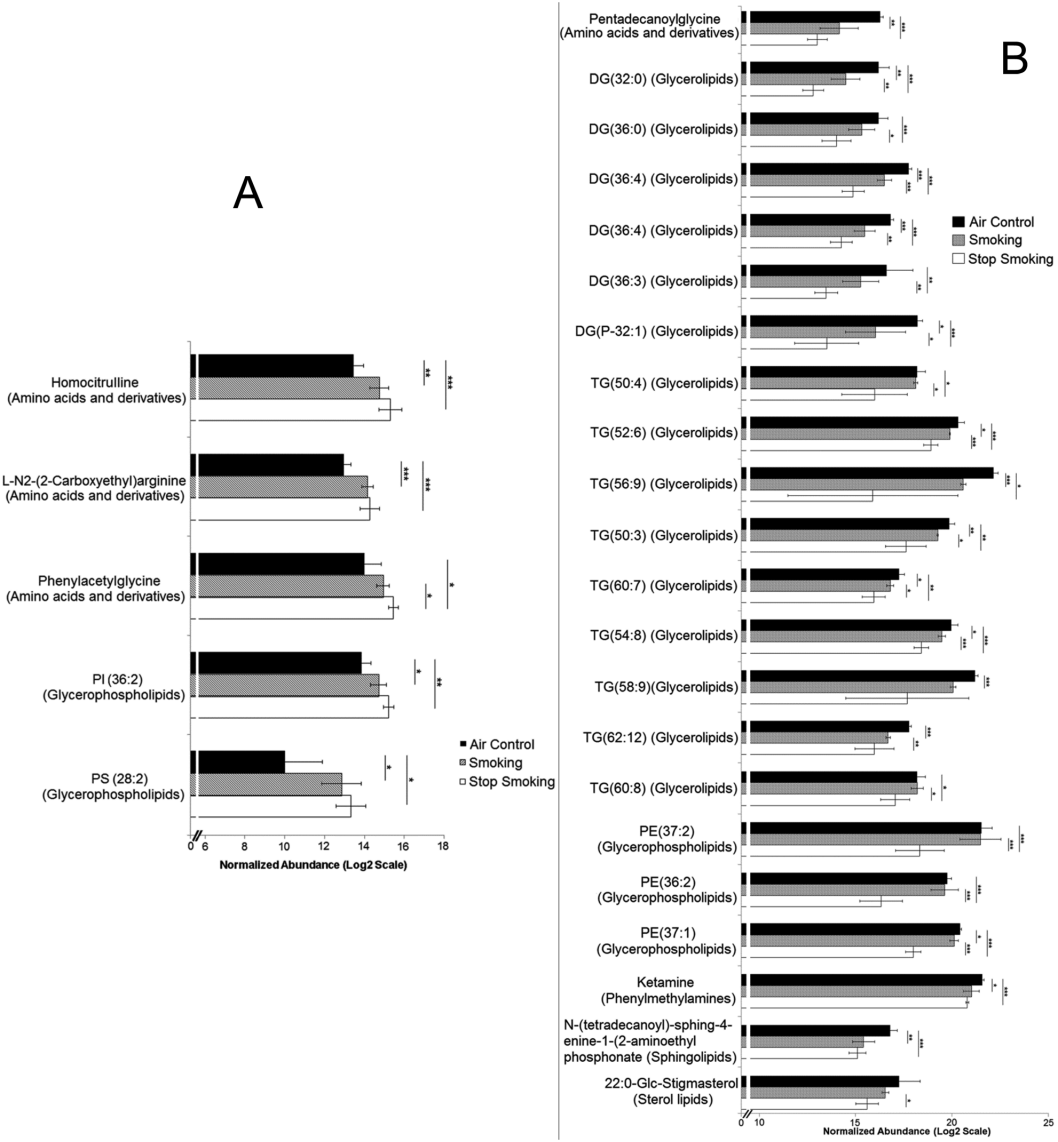
Metabolites which (A) persistently increased and (B) persistently decreased with smoking. In panel A, these metabolites continued increasing even after smoking cessation. In panel B, these metabolites continued decreasing even after smoking cessation. Samples and data were analyzed as described in the methods section. ID Browser in-house database is comprised of Metlin, Lipid Maps and HMDB. PIP = phosphatidylinositol phosphate, PS = phosphatidylserine, TG = triglyceride, DG = diglyceride, PE = phosphatidylethanolamine. Fold change ≥1.5; *p*-value≤0.05, x-axis = Log2 normalized abundance scale; Error bars represent 95% confidence; Significance values *≤0.05, **≤0.01, ***≤0.001.

When endogenous metabolites found in plasma of mice exposed for 6 months to cigarette smoking, air control, or those recovering for 2 months following 4 months of smoking (smoking cessation group) were compared, 82 significant metabolite differences were identified between at least one pair of groups. From these metabolites, 64 were initially identified (Figures 2 and 3) based on exact mass and isotope ratio, with the other 18 remaining unidentified (Table S4). While 60% of the metabolites exhibited reversible changes following smoking cessation, the remaining 40% were persistently affected, suggesting sustained pathological and/or adaptive effects of chronic smoking.

#### 2.3 Plasma metabolites reversibly changed by chronic cigarette smoking

Upon examination of the differentially regulated metabolites among the six month control chronic smoking and smoking cessation groups, 18 compounds were significantly decreased by cigarette smoking and then had levels restored similar to controls following cessation (Figure 2A). Of these, 11 metabolites were triglycerides (TG) of which 10 belonged to the glycerolipid biochemical class of compounds, and one identified as a fatty acid ester. In addition to these metabolites, 19 additional compounds were significantly increased by smoking but decreased to air control levels following cessation (Figure 2B); 5 of these were TGs of which all were glycerolipids, 4 were glycerophospholipids, and 2 were sphingolipids. Little is known of the glycerolipid involvement in cigarette smoke-induced pathology, but sphingolipids have been linked to COPD (emphysema). Of these, ceramides in particular have been reportedly up-regulated in the lung and lung cells in response to cigarette smoke [27]–[29]. Their abundance in plasma of smokers or cigarette smoke-exposed animals has not been reported yet. We noted that, of the differentially regulated sphingolipids, ceramide (d18∶1/20∶0) and galabiosylceramide (d18∶1/25∶0) increased with smoking. Interestingly, although their levels decreased with smoking cessation, they remained elevated when compared to air control levels. In addition, among the other lipids differentially regulated by smoking in plasma, we detected LysoPC(18∶0), which is known to play a role in lipid signaling, smooth muscle contraction, and is associated with atherosclerotic and inflammatory lesions [30]. Also detected was PC(38∶3) which was elevated in smokers compared to both air control and CS-cessation groups. Xu et al [16] also reported an elevation of PC(38∶3) in current smokers compared to former smokers and never smokers for their targeted analysis of PC’s in serum in a human cohort. PC’s are involved in cell signaling and are direct substrates of sphingomyelin synthesis which transfer the choline-phosphate head group to a ceramide lipid anchor [31].

#### 2.4 Plasma metabolites persistently changed following smoking cessation

Figure 3 illustrates the metabolites which remained persistently changed following cigarette smoke exposure, despite smoking cessation for 2 months. Five metabolites, including 3 amino acid derivatives and 2 glycerophospholipids, exhibited continued increase in abundance (Figure 3A) despite smoking cessation. Twenty-two metabolites remained decreased despite smoking cessation (Figure 3B), including 3 glycerophospholipids, and 15 glycerolipids. Unlike the majority of TG species listed in Figure 2A which returned to normal levels following smoking cessation, several TG species (detailed in Figure 3B) were found to be persistently decreased in plasma of mice removed from smoking exposure. Six of the ten TGs reversibly decreased were long-chain TGs of 20 carbon chain length while six out of nine TGs persistently decreased were long chain TGs of 18 carbon chain length. It should be noted that the study was limited to 2 months of smoking cessation and that some or all of these compounds may revert to air control levels after longer periods of smoking cessation.

#### 2.5 Unannotated Metabolites

Eighteen additional metabolites were statistically significant and differentially regulated in the 6 month comparison among the air control, smoking and smoking cessation mice. These were present in the aqueous and neutral fractions. Four metabolites were reversibly decreased with smoking, eight were reversibly increased, three were persistently increased, and three were persistently decreased with smoking. These compounds were not identified through database searching. Additional data (m/z, fraction, retention time and regulation) is provided in Table S4.

### 3 Pathway analysis

Metabolites which showed significant differences and could be tentatively identified at MSI level 2 [21], were grouped based on their distinct chemical classes (Table S3). The recurring classes – purines, amino acids, steroids, fatty acid esters, and prenol lipids, and purines - were investigated to determine if distinct metabolic pathway perturbations can be gauged from alterations of plasma metabolites in response to smoking and/or smoking cessation.

#### 3.1 Purine metabolism

**Figure 4:**
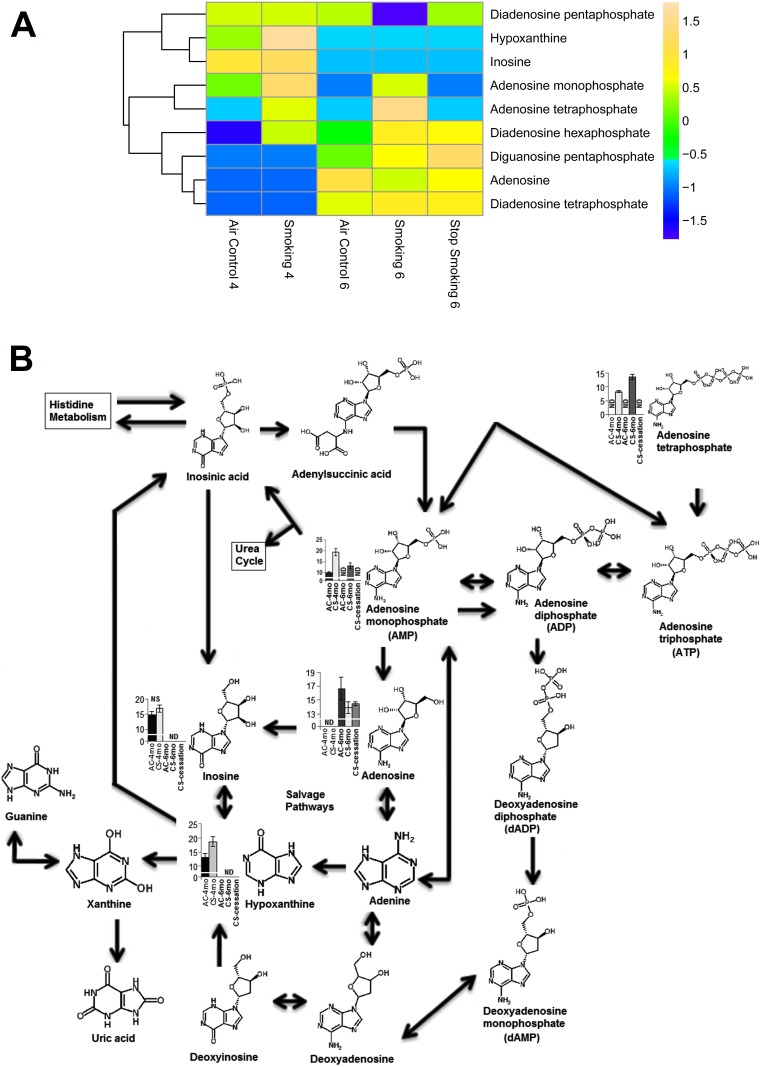
Heat map of (A) purines and (B) purine pathway hits. The mean abundance levels for each of the differentially regulated metabolites detected is shown in the heat map in A. Blue represents a decrease in mean metabolite abundance, and yellow/beige represents an increase in mean metabolite abundance. In panel B, the compounds with indicated abundance levels represent the differentially expressed metabolites identified in the purine metabolism pathway in the current study. Statistical analysis was performed using Mass Profiler Professional software (Agilent) with *p*-value cutoff ≤0.05, NS indicates not significant, ND indicates no data is available, and error bars represent 95% confidence.

Of the nine purine metabolites significantly perturbed by cigarette smoke exposure (*p*≤0.05, fold change ≥1.5) in Figure 4A, five were directly embedded within the purine metabolism pathway (Figure 4B).

Compared to air control exposed groups, cigarette smoke exposure led to increased plasma adenosine monophosphate (AMP) after 4 months (p = 0.046 CS-4mo vs. AC-4mo) and decreased adenosine (p = 0.048 CS-cessation vs. AC-6mo; p = 0.019 CS-6mo vs. AC-6mo). CS exposure also elevated hypoxanthine (p = 0.042 CS-4mo vs. AC-4mo). With continuous smoking (CS-6mo), adenosine tetraphosphate was found elevated (p = 0.0028 CS-6mo vs. CS-4mo) while adenosine monophosphate (AMP) was decreased (p = 0.022 CS-6mo vs. CS4-mo) and inosine was not present after 6 months.

Interestingly, prolonged CS exposure (6mo) also led to the elevations of plasma diguanosine- and diadenosine-polyphosphates, in particular diguanosine pentaphosphate, diadenosine tetraphosphate (p = 0.048 vs. AC-6mo) and diadenosine hexaphosphate (p = 0.050 vs. AC-6mo). Of note, diadenosine pentaphosphate was also present in the plasma of CS-4mo but could not be detected in the CS-6mo group.

The fate and role of purine metabolism in response to cigarette smoking in general, and in COPD in particular remains controversial, perhaps in part because not all studies have followed the same experimental design, but instead covered a range of biological fluids and used multiple detection techniques to study a variety of health conditions. When adenosine was measured in exhaled breath condensate (EBC) its levels, and those of AMP were found to be elevated with increasing COPD severity [32]. Similar to EBC results, Driver et al [33] detected increased levels of adenosine in the BALF of smokers compared to non-smokers, and suggested that their origin may be from plasma extravasation due to epithelial cell injury. In contrast, in sputum, adenosine levels were reportedly lower in smokers with COPD compared to asymptomatic smokers [34], which is a similar trend noted in the murine plasma in our experiments. The short half-life of adenosine (including in plasma) may explain differences in these reports. This underscores the importance of a more global assessment of purine metabolism, which could provide a better understanding of changes induced by short- and long-term exposures to cigarette smoke. Published investigations of other metabolites in this pathway, such as AMP and ATP, detected decreased AMP levels in BALF of COPD smokers compared to control smokers and COPD non-smokers [35] and increased ATP levels in COPD smokers compared to non-smokers [36], whereas no significant differences in ATP levels were measured in EBC among COPD subjects, smokers, and non-smokers [37]. Using a metabolomics approach, we noted significant alterations of AMP and adenosine tetraphosphate in response to smoking, which could be resulting from direct hydrolysis of AMP, ADP, and ATP by cigarette smoke compounds, as suggested by Togna et al [38].

The relevance of changes in diguanosine- and diadenosine-polyphosphates by cigarette smoking is unclear, but it may be important in cardiovascular responses in smokers, since plasma diguanosine pentaphosphate may play a role in blood pressure regulation [39], whereas diadenosine -tetraphosphate, -pentaphosphate, and -hexaphosphate (originally identified in human myocardium [40]) have been associated with eosinophil-related inflammatory and allergic disease and with atherogenesis [24].

While a thorough dissection of the purine metabolism is beyond the scope of our study, our data has to be interpreted in the light that plasma levels of metabolites may reflect a sum of metabolite changes of multiple organs and tissues affected by cigarette smoke. To our knowledge plasma purine levels following cigarette smoking have not been reported in the literature. These results provide novel information linking smoking to perturbed purine metabolism and further studies are being designed to more fully understand their roles in the lung.

#### 3.2 Amino acid metabolism

**Figure 5:**
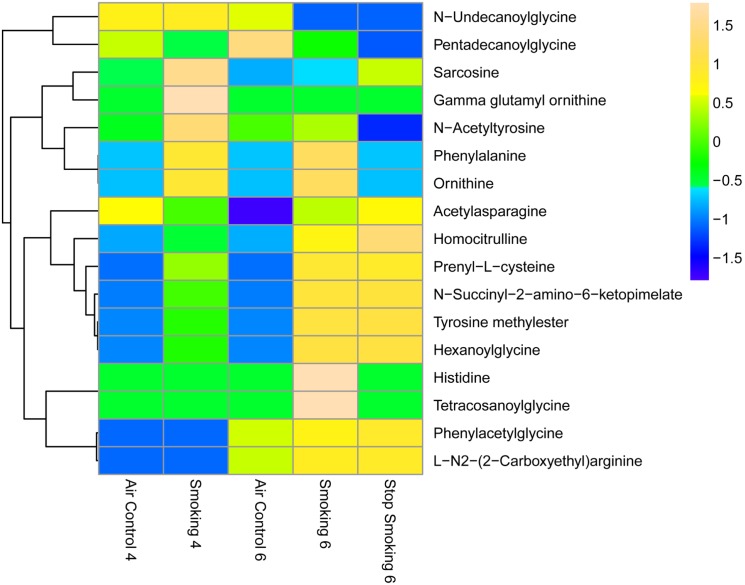
Heat map of differentially regulated amino acids and derivatives. Statistical analysis was performed using Mass Profiler Professional software (Agilent) with *p*-value cutoff ≤0.05. The mean abundance levels for each of the differentially regulated metabolites detected is shown. Blue represents a decrease in mean metabolite abundance, and yellow/beige represents an increase in mean metabolite abundance.

Several amino acids exhibited significantly different (p≤0.05 and ≥1.5 fold change) plasma levels in mice exposed to cigarette smoking compared to those exposed to ambient air (Figure 5).

Among the plasma amino acids that were significantly increased by chronic cigarette smoke exposure, we putatively identified homocitrulline and phenylacetylglycine, while pentadecanoylglycine and n-acetyltyrosine were significantly decreased. We also noted several amino acids which were found significantly changed in abundance after 6 months when compared to 4 months of cigarette smoke exposure. These were phenylalanine, n-acetylasparagine, n-succinyl-2-amino-6-ketopimelate, tyrosine methylester, ornithine, hexanoylglycine, prenyl-L-cysteine, and sarcosine. In addition histidine and tetracosanoylglycine were present in CS-6mo but were absent or possibly at undetectable concentrations in CS-4mo. N-undecanoylglycine and gamma glutamyl ornithine however were present in CS-4mo but absent in CS-6mo. Tetracosanoylglycine, hexanoylglycine, n-undecanoylglycine, and pentadecanoylglycine are all minor metabolites of fatty acids, while homocitrulline and ornithine are both part of the urea cycle. Therefore, abnormal accumulations of these metabolites during cigarette smoke exposure may indicate marked disruptions not only in the amino acid metabolism, but also in interrelated metabolic pathways, in particular that of fatty acid metabolism.

In a 2012 targeted metabolomics assay, Ubhi et al [41] reported perturbations in amino acid metabolism among patients with COPD, emphysema and cachexia. Serum was analyzed from former smokers, never smokers, COPD Gold stage IV subjects, patients with and without emphysema, and patients with and without cachexia. Interestingly, three of the reported abnormal metabolites reported in this study - sarcosine, phenylalanine, and histidine, overlap with those found in the mouse plasma in our study. The decrease in sarcosine levels in emphysema and cachexia patients compared to controls, was paralleled by a significant decrease in sarcosine levels with continued smoking over time in mouse plasma (p = 0.031). The increased phenylalanine levels in cachexia patients compared to controls, was paralleled by a significant increase in phenylalanine with continued smoking in mouse plasma compared to controls (p = 0.027). Lastly, the increase in histidine levels in emphysema and cachexia subjects compared to controls was paralleled by the presence of histidine in plasma of mice exposed to cigarette smoke versus absence of this amino acid in control mice.

Xu et al [16] performed a targeted metabolomics analysis of amino acids and PC’s and reported an increase in ornithine in current smokers compared to former smokers and never smokers. This is consistent with our findings in our mouse model where we detected an increase in ornithine in the CS-6mo compared to the CS-4mo groups (p = 0.010).

While the biological significance of elevation in phenylacetylglycine levels in the CS-cessation plasma compared to AC-6mo group (p = 0.019) remains unclear, it is interesting that elevations of this metabolite was also reported in the urine of heart failure patients compared to healthy controls [42]. In addition to pointing to disruption in these metabolic pathways by cigarette smoking, these results indicate that the mouse model of cigarette smoking may be used to study in more depth the role of relevant metabolic pathways in COPD.

#### 3.3 Steroid Metabolism

**Figure 6:**
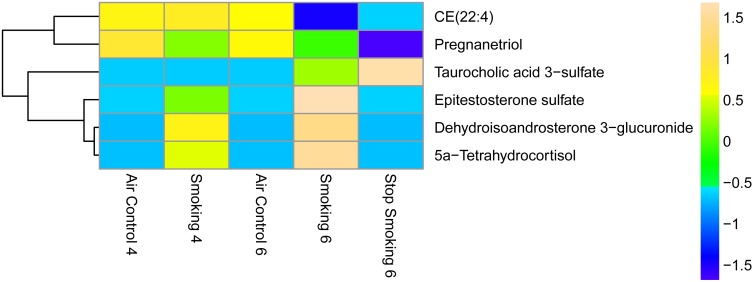
Heat map of differentially regulated steroids and derivatives. Statistical analysis was performed using Mass Profiler Professional software (Agilent) with *p*-value cutoff ≤0.05. The mean abundance levels for each of the differentially regulated metabolites detected is shown. Blue represents a decrease in mean metabolite abundance, and yellow/beige represents an increase in mean metabolite abundance.

We identified six significant steroid metabolites (Figure 6), four of which were upregulated in either the CS-cessation compared to AC-6mo group or higher in CS-6mo compared to CS-4mo. Three of the six steroids (dehydroisoandrosterone 3-glucuronide, CE(22∶4), taurocholic acid 3-sulfate) play a role in bile biosynthesis and secretion. One metabolite, pregnanetriol, was decreased following smoking cessation compared to the air controls. To our knowledge, none of the identified steroids have been reported in the literature in association with cigarette smoking; however, targeted studies have been performed to determine the relationship between other steroids and smoking, with an overall increase in steroid levels observed in smokers compared to non-smokers [43], [44]. English et al [45] reported increased levels of testosterone in the serum of smokers compared to non-smokers, but found no difference in 17-estradiol between groups. Kapoor and Jones examined the effects of smoking on hormonal levels in humans and reported increased levels of adrenal hormones and parathyroid hormones in smoking groups compared to smoking cessation groups [46]. They also reported that a majority of pituitary hormones were increased in the smoking compared to smoking cessation groups, except for insulin-like growth factor which had decreased levels in smokers. Field et al [43] reported increased serum levels of adrenal steroids in male smokers compared to non-smokers. Our results therefore are in agreement with the published literature and suggest that smoking perturbs steroid hormone levels and significantly alters segments of the steroid metabolism pathway.

#### 3.4 Fatty acid esters and prenol lipids

**Figure 7:**
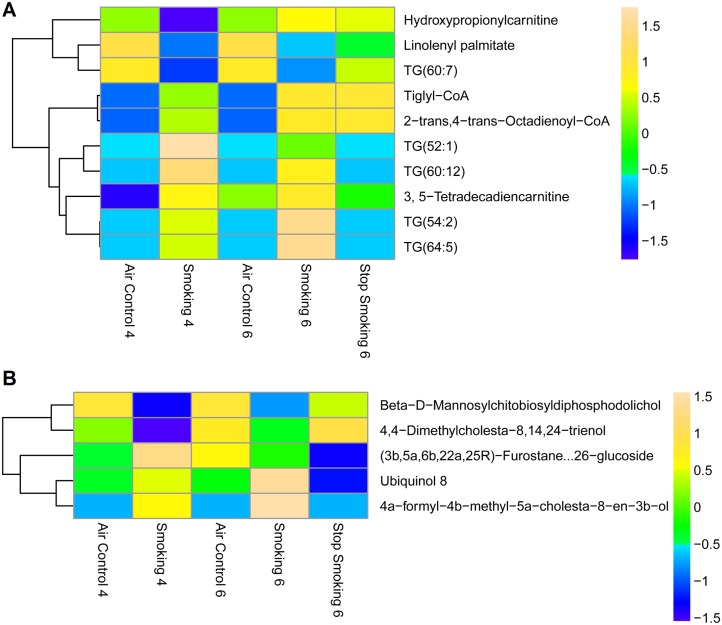
Heat map of differentially regulated (A) fatty acids and (B) prenol lipids. Statistical analysis was performed using Mass Profiler Professional software (Agilent) with *p*-value cutoff ≤0.05. The mean abundance levels for each of the differentially regulated metabolites detected is shown. Blue represents a decrease in mean metabolite abundance, and yellow/beige represents an increase in mean metabolite abundance.

**Figure 8:**
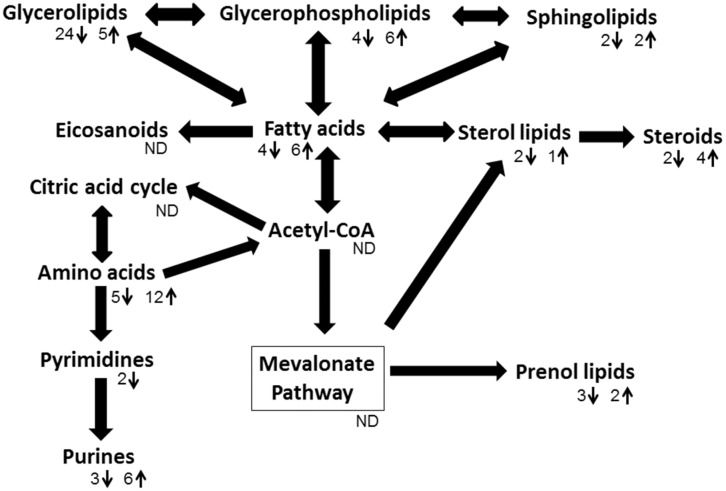
Inter-relation of pathways. Biological relationships among chemical classes of compounds showing the regulation of one group of metabolites and its effects on other biological classes down- or up-stream. Pathway was adapted from KEGG, Lipid Maps, and SMPDB. Metabolites within this pathway passed p-value≤0.05 and fold change ≥1.5 filters. ↓ indicates the number of differentially regulated metabolites which decreased due to smoking, ↑indicates the number of differentially regulated metabolites which increased due to smoking for a particular chemical class of compounds, ^ND^no data was available for these compounds in this dataset.

Six out of the ten fatty acid esters (Figure 7A) identified in the mouse plasma were significantly elevated in the CS-6mo compared to air control group, or elevated in the CS-6mo compared to the CS-4mo. Three of those upregulated in CS-6mo compared to CS-4mo were triglycerides. Two of the fatty acid esters that were up-regulated in the CS-6mo group compared to the AC-6mo group were carnitines. Carnitines are synthesized from the amino acids methionine and lysine [47], [48], and are needed for transport of fatty acids during lipid breakdown for energy production [48], demonstrating the interplay among amino acids, fatty acids, and lipids in metabolism. Figure 8 illustrates the complexity of the regulation of fatty acid levels, which depend on other metabolite groups such as acetyl-CoA and lipids, in addition to rates of synthesis and degradation. Our data suggests that a direct link exists between fatty acid esters metabolism and cigarette smoke exposure, with fatty acids and sterol lipids both increased following smoking. However, the role of fatty acid esters accumulation in response to cigarette smoke remains unclear, especially in light of reports of anti-inflammatory protective effects of fatty acids in COPD [49] as opposed to pro-inflammatory effects of fatty acids in asthma [50].

Prenol lipids (Figure 7B) were predominantly down-regulated by cigarette smoke exposure. It is possible that the equilibrium between acetyl-CoA and the mevalonate pathway becomes unstable and a shift occurs either toward sterol lipid and steroid metabolism, or fatty acid metabolism where we observed increased levels due to smoking (Figure 8). To our knowledge, alternations of prenol lipid plasma concentrations in response to cigarette smoking have not been reported in the literature and may provide a new target for investigation of analysis of smoking related conditions.

#### 3.5 Additional significant metabolites

Additional metabolites were found to be differentially regulated; however, these metabolites are not included in this discussion for several reasons. For example, we did not include compounds that we could not identify through database searching. In addition, some compounds were “orphan” compounds: i.e. the only one present within a specific chemical class. Conversely, the compound may have been annotated as a small peptide and additional analysis was not possible. Finally, because our identifications are tentative, we had increased confidence when multiple compounds mapped to the same pathway. Further information related to these compounds is available at the end of Table S1.

## Conclusion

Chronic cigarette smoke exposure increased plasma levels of glycerophospholipids, amino acids, fatty acid esters, purines, and steroids, and decreased prenol lipid, sterol lipid and glycerolipid levels. Sixty percent of the differentially regulated plasma metabolites reversibly changed following smoking cessation while 40% were persistently altered. Our results highlight profound and sustained metabolic changes in plasma, which may reflect systemic changes induced by smoking in various organs. In addition to the 144 annotated metabolites discussed, an additional seventy-eight differentially regulated metabolites were detected, but remain unannotated when matched against MS databases for accurate mass (Table S4). Refinement in the confirmation of metabolite identification following metabolomics detection will provide a new approach to identify unsuspected pathogenetic pathways and biomarkers associated with COPD and its systemic comorbidities. These, in turn can pave the way for identifying molecular targets which can be used to develop novel therapeutics for smoke-induced health conditions.

## Supporting Information

Figure S1 **Mean linear intercepts (MLI).** MLI in DBA2/J mice exposed to ambient air control (AC) or cigarette smoke (CS), as detailed in the methods section, for 4 months (Mean +SEM; p<0.05; Student’s t test; n = 5 AC and n = 4 CS).
(TIF)

Table S1 **Metabolite Annotations.** Annotated metabolites which passed statistical and fold change analyses in the smoking comparisons in mouse plasma.
(DOCX)

Table S2 **Mass Spectra of Annotated Metabolites.** Selected metabolites to demonstrate the experimental vs theoretical isotopic ratios following database searches.
(DOCX)

Table S3 **Detailed Metabolite Comparisons for Compound Classes.**
(DOCX)

Table S4 **Unannotated Metabolites.** Metabolites which passed statistical and fold change analysis but remained unidentified based on MS library searches.
(DOCX)
