# Supplementary figures and images for: Transient and Persistent Metabolomic Changes in Plasma following Chronic Cigarette Smoke Exposure in a Mouse Model
Source: PLoS One. 2014 Jul 9;9(7):e101855. doi: 10.1371/journal.pone.0101855 (PMC4090193; doi:10.1371/journal.pone.0101855)

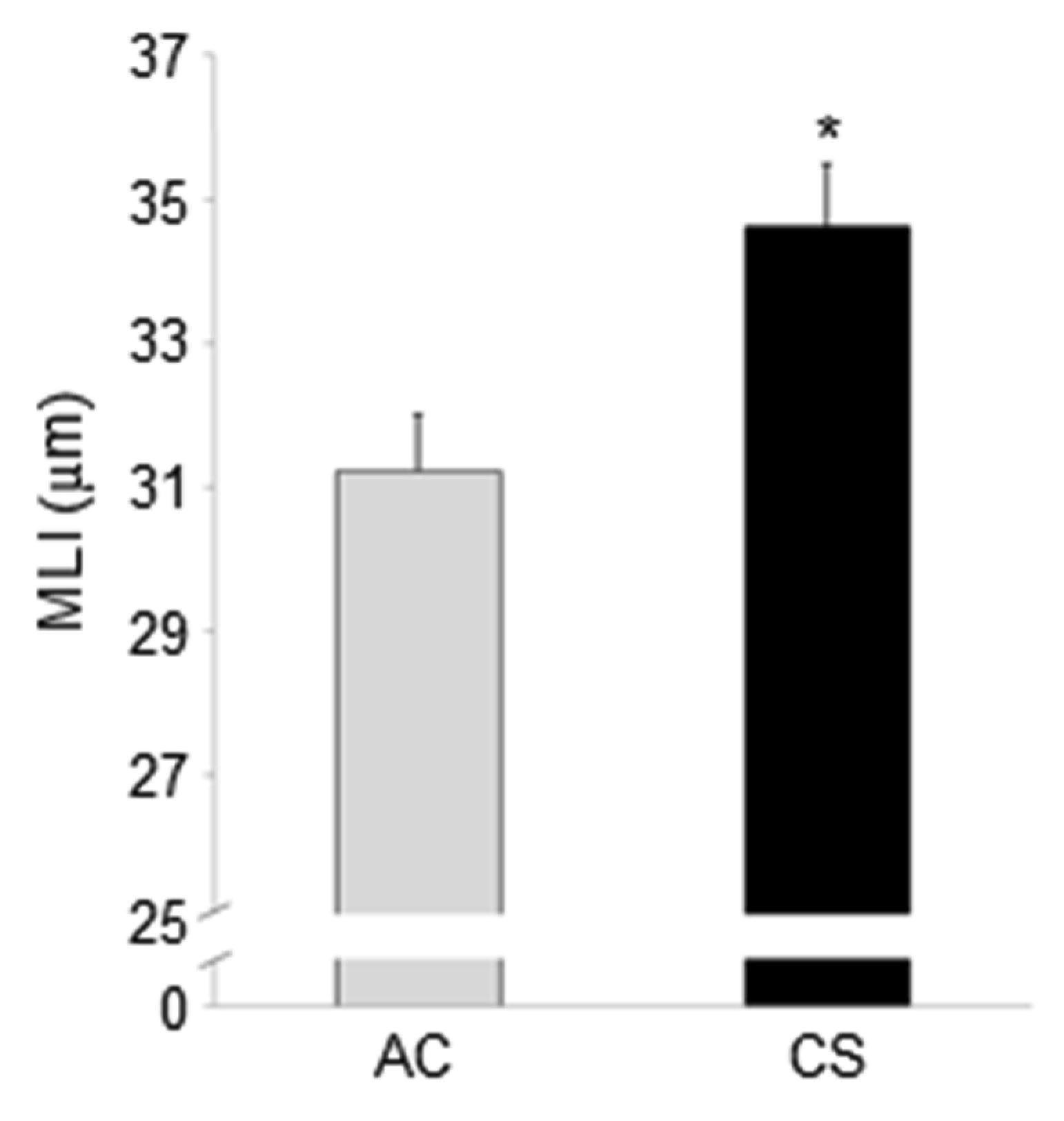

Supplement: Figure S1 — Mean linear intercepts (MLI). MLI in DBA2/J mice exposed to ambient air control (AC) or cigarette smoke (CS), as detailed in the methods section, for 4 months (Mean +SEM; p<0.05; Student’s t test; n = 5 AC and n = 4 CS). (TIF) [file pone.0101855.s001.tif]
