# Supplementary material for: Transient and Persistent Metabolomic Changes in Plasma following Chronic Cigarette Smoke Exposure in a Mouse Model
Source: PLoS One. 2014 Jul 9;9(7):e101855. doi: 10.1371/journal.pone.0101855 (PMC4090193; doi:10.1371/journal.pone.0101855)
Supplement: Table S1 — Metabolite Annotations. Annotated metabolites which passed statistical and fold change analyses in the smoking comparisons in mouse plasma. (DOCX) [file pone.0101855.s002.docx]

**Supplemental Table 1**: Annotated metabolites which passed FDR < 0.05 statistical significance and fold change > 1.5 analysis in the smoking comparisons in mouse plasma. All annotations are MSI level 2, based on chemical properties as a result of selective solvent extraction, exact mass, and spectral isotope matches to database libraries.

| **Metabolite** | **Formula** | **Fraction Detected** | **Retention Time (min)** | **Mass Accuracy (ppm)** |
| --- | --- | --- | --- | --- |
| (3b,5a,6b,22a,25R)-Furostane-22-methoxy-3,6,26-triol 3-[glucosyl-(1,2)-[xylosyl-(1,3)]-glucosyl-(1,4)-galactoside] 26-glucoside | C57 H96 O29 | Neutral | 7.838 | 6.4 |
| 1-(O-alpha-D-glucopyranosyl)-29-keto-(1,3R,31R)-dotriacontanetriol | C38 H74 O9 | Neutral | 6.344 | 1.2 |
| 12-Oxo-20-carboxy-leukotriene B4 | C20 H28 O6 | Aqueous | 6.230 | 4.9 |
| 16:1-Glc-Cholesterol | C49 H84 O7 | Neutral | 7.697 | 1.2 |
| 22:0-Glc-Stigmasterol | C57 H100 O7 | Neutral | 9.922 | 1.4 |
| 22:1-Glc-Sitosterol | C57 H100 O7 | Neutral | 9.96 | 0.69 |
| 2-trans,4-trans-Octadienoyl-CoA | C29 H46 N7 O17 P3 S | Aqueous | 5.827 | 0.92 |
| 3-(4-hydroxy-1-naphthoxy)lactic acid | C13 H12 O5 | Aqueous | 5.959 | 9.1 |
| 3, 5-Tetradecadiencarnitine | C21 H37 N O4 | Phospholipid | 6.844 | 4.2 |
| 3-Methylindole | C9 H9 N | Aqueous | 2.671 | 7.8 |
| 4-(3-Pyridyl)-butanoic acid | C9 H11 N O2 | Aqueous | 6.484 | 1.6 |
| 4,4-Dimethylcholesta-8,14,24-trienol | C29 H46 O | Aqueous | 6.636 | 6.9 |
| 4-Hydroxy-4-(3-pyridyl)-butanoic acid | C9 H11 N O3 | Aqueous | 1.111 | 3.8 |
| 4-Oxo-4-(3-pyridyl)-butanoic acid | C9 H9 N O3 | Aqueous | 4.980 | 0.49 |
| 4α-formyl-4β-methyl-5α-cholesta-8-en-3β-ol | C29 H48 O2 | Neutral | 7.039 | 2.1 |
| 5a-Tetrahydrocortisol | C21 H34 O5 | Aqueous | 5.944 | 3.1 |
| 6-Hydroxyketamine | C13 H16 Cl N O2 | Neutral | 0.981 | 4.9 |
| β-1,4-D-Mannosylchitobiosyldiphosphodolichol | C47 H82 N2 O22 P2 | Aqueous | 15.186 | 9.1 |
| Acetylasparagine | C6 H10 N2 O4 | Aqueous | 2.801 | 6.0 |
| Adenosine | C10 H13 N5 O4 | Aqueous | 2.095 | 0.03 |
| Adenosine monophosphate | C10 H14 N5 O7 P | Aqueous | 1.002 | 3.9 |
| Adenosine tetraphosphate | C10 H17 N5 O16 P4 | Aqueous | 6.495 | 4.4 |
| CDP-DG(40:4) | C52 H89 N3 O15 P2 | Neutral | 10.058 | 6.6 |
| CE(22:4) | C49 H80 O2 | Aqueous | 12.646 | 6.8 |
| Ceramide (d18:1/20:0) | C38 H75 N O3 | Aqueous | 6.318 | 1.6 |
| Cotinine methonium ion | C11 H15 N2 O | Aqueous | 6.460 | 5.6 |
| Cytidine diphosphate (CDP) | C9 H15 N3 O11 P2 | Aqueous | 6.340 | 2.9 |
| Dehydroisoandrosterone 3-glucuronide | C25 H36 O8 | Aqueous | 0.793 | 0.81 |
| DG(32:0) | C35 H68 O5 | Neutral | 8.137 | 0.98 |
| DG(36:0) | C39 H76 O5 | Neutral | 10.775 | 4.2 |
| DG(36:3) | C39 H70 O5 | Neutral | 8.192 | 0.71 |
| DG(36:4) | C39 H68 O5 | Neutral | 7.994 | 3.3 |
| DG(36:4) | C39 H68 O5 | Neutral | 8.000 | 3.3 |
| DG(P-32:1) | C35 H66 O4 | Neutral | 10.333 | 0.02 |
| Diadenosine hexaphosphate | C20 H30 N10 O25 P6 | Neutral | 11.268 | 0.91 |
| Diadenosine pentaphosphate | C20 H29 N10 O22 P5 | Aqueous | 0.719 | 2.4 |
| Diadenosine tetraphosphate | C20 H28 N10 O19 P4 | Aqueous | 6.063 | 3.2 |
| Diguanosine pentaphosphate | C20 H29 N10 O24 P5 | Aqueous | 0.888 | 5.6 |
| Dihydrocaffeic acid 3-O-glucuronide | C15 H18 O10 | Aqueous | 3.739 | 0.56 |
| Epitestosterone sulfate | C19 H28 O5 S | Aqueous | 5.606 | 0.08 |
| Gamma glutamyl ornithine | C10 H19 N3 O5 | Phospholipid | 4.930 | 1.7 |
| Galabiosylceramide | C55 H105 N O13 | Neutral | 9.969 | 6.5 |
| Hexanoylglycine | C8 H15 N O3 | Aqueous | 5.960 | 6.5 |
| Histidine | C6 H9 N3 O2 | Aqueous | 0.761 | 6.4 |
| Homocitrulline | C7 H15 N3 O3 | Aqueous | 1.064 | 7.3 |
| Hydroxypropionylcarnitine | C10 H19 N O5 | Aqueous | 5.575 | 3.6 |
| Hypoxanthine | C5 H4 N4 O | Aqueous | 1.709 | 0.31 |
| Inosine | C10 H12 N4 O5 | Aqueous | 1.971 | 0.81 |
| Ketamine | C13 H16 Cl N O | Aqueous | 6.234 | 0.62 |
| Linolenyl palmitate | C34 H62 O2 | Neutral | 7.230 | 7.2 |
| L-N2-(2-Carboxyethyl)arginine | C9 H18 N4 O4 | Aqueous | 1.769 | 0.91 |
| LysoPC(18:0) | C26 H54 N O7 P | Phospholipid | 5.181 | 2.8 |
| N-(tetradecanoyl)-sphing-4-enine-1-(2-aminoethylphosphonate) | C34 H69 N2 O5 P | Neutral | 7.980 | 6.7 |
| N-Acetyltyrosine | C11 H13 N O4 | Aqueous | 5.651 | 5.2 |
| Nicotine glucuronide | C16 H22 N2 O6 | Aqueous | 1.800 | 6.7 |
| Nicotine isomethonium ion | C11 H17 N2 | Aqueous | 15.224 | 5.1 |
| Nicotine-delta 1'(5')-iminium ion | C10 H13 N2 | Aqueous | 1.777 | 4.4 |
| Nicotyrine | C10 H10 N2 | Aqueous | 2.687 | 9.4 |
| N-Succinyl-2-amino-6-ketopimelate | C11 H15 N O8 | Aqueous | 0.750 | 2.4 |
| N-Undecanoylglycine | C13 H25 N O3 | Neutral | 1.055 | 1.3 |
| Ornithine | C5 H12 N2 O2 | Aqueous | 0.764 | 6.1 |
| PA(42:3) | C45 H83 O8 P | Phospholipid | 7.781 | 0.12 |
| PC(38:3) | C46 H86 N O8 P | Phospholipid | 8.019 | 4.3 |
| PE(36:2) | C41 H78 N O8 P | Phospholipid | 7.279 | 1.6 |
| PE(37:1) | C42 H82 N O8 P | Neutral | 7.967 | 2.7 |
| PE(37:2) | C42 H80 N O8 P | Phospholipid | 7.545 | 0.62 |
| Pentadecanoylglycine | C17 H33 N O3 | Neutral | 8.013 | 1.3 |
| Phenylacetylglycine | C10 H11 N O3 | Aqueous | 5.841 | 2.9 |
| Phenylalanine | C9 H11 N O2 | Aqueous | 6.482 | 1.6 |
| PI(36:2) | C45 H83 O13 P | Aqueous | 6.659 | 6.7 |
| Pregnanetriol | C21 H36 O3 | Neutral | 7.973 | 1.6 |
| Prenyl-L-cysteine | C8 H15 N O2 S | Aqueous | 6.097 | 3.6 |
| PS(27:0) | C33 H64 N O10 P | Neutral | 4.178 | 1.4 |
| PS(28:2) | C34 H62 N O10 P | Aqueous | 6.798 | 2.4 |
| Pyrrolidine | C4 H9 N | Aqueous | 0.809 | 2.6 |
| Sarcosine | C3 H7 N O2 | Aqueous | 0.804 | 2.0 |
| Taurocholic acid 3-sulfate | C26 H45 N O10 S2 | Aqueous | 6.005 | 5.9 |
| Tetracosanoylglycine | C26 H51 N O3 | Phospholipid | 6.749 | 5.4 |
| TG(50:3) | C53 H96 O6 | Neutral | 10.076 | 2.5 |
| TG(50:4) | C53 H94 O6 | Neutral | 9.995 | 0.33 |
| TG(52:1) | C55 H104 O6 | Aqueous | 6.346 | 2.8 |
| TG(52:6) | C55 H94 O6 | Neutral | 9.975 | 1.1 |
| TG(54:2) | C57 H106 O6 | Neutral | 10.717 | 1.6 |
| TG(54:6) | C57 H98 O6 | Neutral | 10.280 | 5.9 |
| TG(54:8) | C57 H94 O6 | Neutral | 9.891 | 6.0 |
| TG(56:2) | C59 H110 O6 | Neutral | 10.908 | 2.7 |
| TG(56:8) | C59 H98 O6 | Neutral | 9.974 | 3.7 |
| TG(56:8) | C59 H98 O6 | Neutral | 10.082 | 4.3 |
| TG(56:9) | C59 H96 O6 | Neutral | 9.935 | 4.2 |
| TG(58:10) | C61 H98 O6 | Phospholipid | 10.115 | 7.0 |
| TG(58:10) | C61 H98 O6 | Neutral | 9.957 | 3.8 |
| TG(58:11) | C61 H96 O6 | Neutral | 9.948 | 1.8 |
| TG(58:7) | C61 H104 O6 | Neutral | 10.600 | 1.9 |
| TG(58:9) | C61 H100 O6 | Phospholipid | 10.424 | 0.67 |
| TG(60:11) | C63 H100 O6 | Neutral | 9.979 | 1.7 |
| TG(60:12) | C63 H98 O6 | Neutral | 10.031 | 0.30 |
| TG(60:12) | C63 H98 O6 | Neutral | 9.902 | 4.0 |
| TG(60:12) | C63 H98 O6 | Neutral | 9.946 | 2.9 |
| TG(60:7) | C62 H108 O5 | Neutral | 10.022 | 4.6 |
| TG(60:7) | C63 H108 O6 | Neutral | 10.593 | 0.74 |
| TG(60:8) | C63 H106 O6 | Neutral | 10.653 | 3.2 |
| TG(61:7) | C64 H110 O6 | Neutral | 11.091 | 7.4 |
| TG(62:12) | C65 H102 O6 | Neutral | 10.212 | 2.9 |
| TG(62:13) | C65 H100 O6 | Neutral | 9.990 | 0.30 |
| TG(62:14) | C65 H98 O6 | Neutral | 9.900 | 1.6 |
| TG(62:14) | C65 H98 O6 | Neutral | 9.928 | 2.3 |
| TG(62:4) | C65 H118 O6 | Neutral | 11.138 | 1.0 |
| TG(64:14) | C67 H102 O6 | Neutral | 10.020 | 1.0 |
| TG(64:5) | C67 H122 O5 | Aqueous | 14.788 | 2.9 |
| Tiglyl-CoA | C26 H42 N7 O17 P3 S | Aqueous | 7.607 | 8.6 |
| Tyrosine methylester | C10 H13 N O3 | Aqueous | 5.966 | 6.9 |
| Ubiquinol 8 | C49 H78 O4 | Neutral | 7.064 | 2.5 |

*Below*: Additional annotated metabolites which passed p-value (≤0.05) and fold change (≥1.5) filters but only 1-3 were unique to a chemical class, or the annotations were peptides which were beyond the scope of the manuscript.

| **Metabolite** | **Formula** | **Fraction Detected** | **Retention Time (min)** | **Mass Accuracy (ppm)** | **Chemical Class** |
| --- | --- | --- | --- | --- | --- |
| Neurine | C5 H13 N O | Phospholipid | 0.651 | 7.3 | Alkaloids and Derivatives |
| Zileuton | C11 H12 N2 O2 S | Neutral lipid | 0.981 | 3.3 | Benzothiophenes |
| Uracil | C4 H4 N2 O2 | Aqueous | 3.929 | 0.065 | Diazines |
| Arachidyl alcohol | C20 H4 2O | Aqueous | 6.094 | 4.5 | Fatty Alcohols |
| Palmitaldehyde | C16 H32 O | Phospholipid | 6.441 | 0.93 | Fatty Aldehydes |
| Gossypetin 8-glucuronide-3-sulfate | C21 H18 O17 S | Phospholipid | 0.661 | 3.9 | Flavonoids |
| Indoleacrylic acid | C11 H9 N O2 | Aqueous | 2.684 | 0.24 | Indoles |
| 1H-Indole-3-carboxaldehyde | C9 H7 N O | Aqueous | 2.677 | 1.64 | Indoles |
| Isopropyl citrate | C10 H18 O7 | Aqueous | 6.453 | 7.3 | Keto-Acids and Derivatives |
| Trimethylamine N-oxide | C3 H9 N O | Aqueous | 0.751 | 5.3 | Organic Oxoazanium Compounds |
| Methionyl-Phenylalanine | C14 H20 N2 O3 S | Aqueous | 5.99 | 0.46 | Peptides |
| Tyrosyl-Valine | C14 H20 N2 O4 | Aqueous | 1.78 | 2.2 | Peptides |
| L-prolyl-L-proline | C10 H16 N2 O3 | Aqueous | 6.415 | 0.86 | Peptides |
| Threoninyl-Arginine | C10 H21 N5 O4 | Aqueous | 5.828 | 4.8 | Peptides |
| Somatostatin | C72 H98 N16 O17 S2 | Aqueous | 6.238 | 3.8 | Peptides |
| Methionyl-Serine | C8 H16 N2 O4 S | Aqueous | 1.682 | 3.7 | Peptides |
| Tyrosyl-Hydroxyproline | C14 H18 N2 O5 | Aqueous | 3.747 | 1.0 | Peptides |
| Threoninyl-Threonine | C8 H16 N2 O5 | Neutral lipid | 1.933 | 9.9 | Peptides |
| Histidinyl-Histidine | C12 H16 N6 O3 | Aqueous | 0.904 | 1.8 | Peptides |
| Hydroxyprolyl-Tryptophan | C16 H19 N3 O4 | Neutral | 1.733 | 6.0 | Peptides |
| Kinetensin 1-7 | C41 H65 N15 O9 | Aqueous | 6.658 | 9.2 | Peptides |
| Methionyl-Glycine | C7 H14 N2 O3 S | Aqueous | 9.063 | 3.4 | Peptides |
| Glutaminyl-Histidine | C11 H17 N5 O4 | Aqueous | 0.764 | 2.2 | Peptides |
| Leucyl-Leucine | C12 H24 N2 O3 | Neutral lipid | 0.954 | 3.5 | Peptides |
| S-Nitrosoglutathione | C10 H16 N4 O7 S | Aqueous | 1.076 | 5.1 | Peptides |
| Isoleucyl-Isoleucine | C12 H24 N2 O3 | Aqueous | 12.774 | 2.5 | Peptides |
| Ganglioside GQ1c (d18:0/14:0) | C103 H177 N5 O55 | Neutral lipid | 8.218 | 2.0 | Polysaccharides |
| 2-Amino-4-oxo-6-(1',2',3'-trihydroxypropyl)-diquinoid-7,8-dihydroxypterin | C9 H15 N5 O6 | Aqueous | 6.127 | 4.5 | Pteridines and Derivatives |
| Uridine diphosphate-N-acetylglucosamine | C17 H27 N3 O17 P2 | Aqueous | 6.061 | 6.0 | Pyrimidine Nucleotides |
| 3-O-Sulfogalactosylceramide (d18:1/18:0) | C42 H81 N O11 S | Neutral lipid | 5.512 | 4.9 | Sphingolipids |
| 3-Indole carboxylic acid glucuronide | C15 H15 N O8 | Aqueous | 6.119 | 0.62 | Sugar Acids and Derivatives |
| N-(1-Deoxy-1-fructosyl)valine | C11 H21 N O7 | Aqueous | 0.761 | 2.48 | Trisaccharides |
