# Supplementary material for: Transient and Persistent Metabolomic Changes in Plasma following Chronic Cigarette Smoke Exposure in a Mouse Model
Source: PLoS One. 2014 Jul 9;9(7):e101855. doi: 10.1371/journal.pone.0101855 (PMC4090193; doi:10.1371/journal.pone.0101855)
Supplement: Table S2 — Mass Spectra of Annotated Metabolites. Selected metabolites to demonstrate the experimental vs theoretical isotopic ratios following database searches. (DOCX) [file pone.0101855.s003.docx]

**Supplemental Table 2:** Selected Metabolite Annotations

**Part A**: MS database annotations. Each experimental isotopic peak in black is surrounded by a red box which indicates the theoretical isotopic ratio.

**4-hydroxy-4-(3-pyridyl)-butanoic acid**; Database score = 97.14; Mass error = 3.78ppm

**4-(3-pyridyl)-butanoic acid**; Database score = 90.10; Mass error = 1.58ppm

**3-methylindole**; Database score = 92.55; Mass error = 7.85ppm

**Pyrrolidine**; Database score = 83.33; Mass error = 2.56ppm

**Adenosine tetraphosphate**; Database score = 72.88; Mass error = 4.4ppm

**Adenosine monophosphate**; Database score = 87.04; Mass error = 3.88ppm

**Inosine**; Database score = 95.55; Mass error = 0.81ppm

**Hypoxanthine**; Database score = 97.26; Mass error = 0.31ppm

**Histidine**; Database score = 80.5; Mass Error = 6.44ppm

**Homocitrulline**; Database score = 78.74; Mass error = 7.36ppm

**Sarcosine**; Database score = 89.17; Mass error = 2.04ppm

**Phenylalanine;** Database score = 88.28; Mass error = 1.58ppm

**Ketamine**; Database score = 98; Mass error = 0.62ppm

**6-Hydroxyketamine**; Database score = 85.39; Mass error = 4.87ppm

**Pregnanetriol**; Database score = 97.76; Mass error = 1.56ppm

**1-(O-alpha-D-glucopyranosyl)-29-keto-(1,3R,31R)-dotriacontanetriol**; Database score = 92.38;

Mass error = 1.2ppm

**N-Undecanoylglycine**; Database score = 85.8; Mass error = 1.26ppm

**4alpha-formyl-4beta-methyl-5alpha-cholesta-8-en-3beta-ol**; Database score = 93.26;

Mass error = 2.06ppm

**TG(18:1/20:2/22:4)** (*Note: Adduct = M+Na, not M+H*); Database score = 71; Mass error = 0.62ppm

**TG(16:0/18:2/20:0))**; Database score = 88.82; Mass error = 1.6ppm

**TG(18:1/18:1/20:0)** (*Note: Adduct = M+Na, not M+H*); Database score = 75.08; Mass error = 2.69ppm

**PS(14:0/13:0)**; Database score = 93.93; Mass error = 1.38ppm

**PE(22:2/15:0)**; Database score = 77.4; Mass error = 0.619ppm

**PE(18:1/19:0)**; Database score = 91.1, Mass error = 2.67ppm

**PE(16:0/20:2)**; Database score = 79.79, Mass error = 4.95ppm

**DG(P-14:0/18:1)**; Database score = 86.97; Msss error = 0.018ppm

**DG(16:0/20:4/0:0)**; (*Note: Adduct = M+NH_4_, not M+H*); Database score = 89.27; Mass error = 4.2ppm

**DG(22:2/14:1/0:0)** (*Note: Adduct = M+NH_4_, not M+H*); Database score = 79.23; Mass error = 0.707ppm

**LysoPC(18:0)**; Database score = 88.31; Mass error = 2.82ppm

**Ubiquinol 8**: Database score = 86.97; Mass error = 2.51ppm
