# Supplementary material for: Transient and Persistent Metabolomic Changes in Plasma following Chronic Cigarette Smoke Exposure in a Mouse Model
Source: PLoS One. 2014 Jul 9;9(7):e101855. doi: 10.1371/journal.pone.0101855 (PMC4090193; doi:10.1371/journal.pone.0101855)
Supplement: Table S3 — Detailed Metabolite Comparisons for Compound Classes. (DOCX) [file pone.0101855.s004.docx]

**Supplemental Table 3**: Detailed Metabolite Comparisons for each Compound Class

Tables 2-6 below reflect the more recurrent chemical classes detected and identified as significant. These complement the heat map figures in the manuscript. Statistical analysis (fold change ≥ 1.5, p-value ≤ 0.05) was performed using Mass Profiler Professional software (Agilent Technologies). All annotations are MSI level 2, based on chemical properties as a result of selective solvent extraction, exact mass, and spectral isotope matches to database libraries. Identifications were performed using ID Browser which is comprised of HMDB, Lipid Maps, and Metlin databases.

| **Cigarette smoke metabolites** | **Regulation(s) and Comparison(s)** | ***p*-value** |
| --- | --- | --- |
| 3-Methylindole | Up in CS-6mo compared to AC-6mo | 0.004432 |
|  | Up in CS-cessation compared to AC-6mo | 0.007307 |
|  | Down in CS-cessation compared to CS-6mo | 0.7691 |
| 4-(3-Pyridyl)-butanoic acid | Up in CS-6mo compared to AC-6mo | 0.001702 |
|  | Up in CS-cessation compared to AC-6mo | 0.001163 |
|  | Up in CS-cessation compared to CS-6mo | 0.1467 |
| 4-Hydroxy-4-(3-pyridyl)-butanoic acid | Up in CS-4mo compared to AC-4mo | 0.4718 |
|  | Up in CS-6mo compared to AC-6mo | 0.002738 |
|  | Up in CS-cessation compared to AC-6mo | 0.000698 |
|  | Down in CS-cessation compared to CS-6mo | 0.3230 |
| 4-Oxo-4-(3-pyridyl)-butanoic acid | Up in CS-6mo compared to AC-6mo | 0.4026 |
|  | Up in CS-cessation compared to AC-6mo | 0.4702 |
|  | Down in CS-cessation compared to CS-6mo | 0.1735 |
| Cotinine methonium ion | Up in CS-6mo compared to AC-6mo | 0.01496 |
|  | Up in CS-cessation compared to AC-6mo | 0.000940 |
|  | Up in CS-cessation compared to CS-6mo | 0.8195 |
| Nicotine glucuronide | Up in CS-6mo compared to AC-6mo | 0.000433 |
|  | Up in CS-cessation compared to AC-6mo | 0.02691 |
|  | Down in CS-cessation compared to CS-6mo | 0.08352 |
| Nicotine-delta 1'(5')-iminium ion | Up in CS-4mo compared to AC-4mo | 0.009388 |
| Nicotine isomethonium ion | Up in CS-6mo compared to AC-6mo | 0.2382 |
|  | Down in CS-cessation compared to AC-6mo | 0.6229 |
|  | Down in CS-cessation compared to CS-6mo | 0.2005 |
| Nicotyrine | Up in CS-6mo compared to AC-6mo | 0.01256 |
|  | Up in CS-cessation compared to AC-6mo | 0.00371 |
|  | Down in CS-cessation compared to CS-6mo | 0.5739 |
| Pyrrolidine* | Up in CS-4mo compared to AC-4mo | 0.000025 |
|  | Up in CS-6mo compared to AC-6mo | 0.02161 |
|  | Up in CS-cessation compared to AC-6mo | 0.05973 |
|  | Down in CS-cessation compared to CS-6mo | 0.6182 |

**Table 1: Cigarette smoke metabolites identified in the aqueous fraction in the plasma of smoking mouse models**. Samples were analyzed on an Agilent 6410 ESI-TOF in positive ionization mode, data was processed using Mass Profiler Professional, and quantitative data was obtained using Mass Hunter Quantitative analysis software. Tentative identification was performed using ID Browser within the Mass Profiler Professional software (Agilent). ID Browser in-house database is comprised of Metlin, Lipid Maps and HMDB. *present in foods as a flavoring ingredient.

| **Purines** | **Regulation(s) and comparison(s)** | ***p*-value** |
| --- | --- | --- |
| Adenosine tetraphosphate | Up in CS-6mo compared to CS-4mo | 0.00279 |
| Diadenosine tetraphosphate | Up in CS-6mo compared to AC-6mo | 0.04773 |
| Diadenosine hexaphosphate | Up in CS-cessation compared to AC-6mo | 0.050 |
|  | Up in CS-6mo compared to AC-6mo | 0.050 |
| Diguanosine pentaphosphate | Present in CS-6mo, absent in CS-4mo | n/a |
| Hypoxanthine | Up in CS-4mo compared to AC-4mo | 0.04212 |
| Adenosine monophosphate | Up in CS-4mo compared to AC-4mo | 0.04598 |
|  | Down in CS-6mo compared to CS-4mo | 0.02216 |
| Adenosine | Down in CS-cessation compared to AC-6mo | 0.04774 |
|  | Down in CS-6mo compared to AC-6mo | 0.01896 |
| Diadenosine pentaphosphate | Present in CS-4mo, absent in CS-6mo | n/a |
| Inosine | Present in CS-4mo, absent in CS-6mo | n/a |

**Table 2: Differentially regulated purine metabolites putatively identified in the plasma of the smoking mouse model**.

| **Amino acids and derivatives** | **Regulation(s) and comparison(s)** | ***p*-value** |
| --- | --- | --- |
| Homocitrulline | Up in CS-cessation compared to AC-6mo | 0.000429 |
|  | Up in CS-6mo compared to AC-6mo | 0.003366 |
| L-N2-(2-Carboxyethyl)arginine | Up in CS-cessation compared to AC-6mo | 8.8x10^-4 |
|  | Up in CS-6mo compared to AC-6mo | 3.8x10^-4 |
| Phenylacetylglycine | Up in CS-cessation compared to AC-6mo | 0.01985 |
| Histidine | Present in CS-6mo; absent in CS-4mo | n/a |
| Tetracosanoylglycine | Present in CS-6mo, absent in CS-4mo | n/a |
| Phenylalanine | Up in CS-6mo compared to CS-4mo | 0.02674 |
| N-Acetylasparagine | Up in CS-6mo compared to CS-4mo | 0.01985 |
|  | Down in CS-4mo compared to AC-4mo | 0.03864 |
| N-Succinyl-2-amino-6-ketopimelate | Up in CS-6mo compared to CS-4mo | 0.02914 |
| Tyrosine methylester | Up in CS-6mo compared to CS-4mo | 0.01766 |
| Ornithine | Up in CS-6mo compared to CS-4mo | 0.01001 |
| Hexanoylglycine | Up in CS-6mo compared to CS-4mo | 0.03409 |
| Prenyl-L-cysteine | Up in CS-6mo compared to CS-4mo | 0.00627 |
| N-acetyltyrosine | Down in CS-cessation compared to AC-6mo | 0.01297 |
| N-Undecanoylglycine | Present in CS-4mo, absent in CS-6mo | n/a |
| Gamma glutamyl ornithine | Present in CS-4mo, absent in CS-6mo | n/a |
| Sarcosine | Down in CS-6mo compared to CS-4mo | 0.03068 |
| Pentadecanoylglycine | Down in CS-cessation compared to AC-6mo | 1.6x10^-6 |
|  | Down in CS-6mo compared to AC-6mo | 0.002832 |

**Table 3: Differentially regulated amino acids and derivatives which were putatively identified in the plasma of the smoking mouse model**.

| **Steroids and steroid derivatives** | **Regulation(s) and comparison(s)** | ***p*-value** |
| --- | --- | --- |
| Epitestosterone sulfate | Up in CS-6mo compared to CS-4mo | 0.00364 |
| Dehydroisoandrosterone 3-glucuronide | Up in CS-6mo compared to CS-4mo | 0.00362 |
| 5a-Tetrahydrocortisol | Up in CS-6mo compared to CS-4mo | 0.01081 |
| Taurocholic acid 3-sulfate | Up in CS-cessation compared to AC-6mo | 0.02745 |
| CE(22:4) | Down in CS-6mo compared to CS-4mo | 0.01163 |
| Pregnanetriol | Down in CS-cessation compared to AC-6mo | 0.00892 |

**Table 4: Differentially regulated steroids and derivatives which were putatively identified in the plasma of the smoking mouse model**.

| **Fatty acid esters** | **Regulation(s) and comparison(s)** | ***p*-value** |
| --- | --- | --- |
| Tiglyl-CoA | Up in CS-6mo compared to CS-4mo | 0.04406 |
| TG(54:2) | Up in CS-6mo compared to CS-4mo | 0.01229 |
| TG(64:5) | Up in CS-6mo compared to CS-4mo | 0.03491 |
| 2-trans,4-trans-Octadienoyl-CoA | Up in CS-6mo compared to CS-4mo | 0.02984 |
| 3, 5-Tetradecadiencarnitine | Up in CS-6mo compared to AC-6mo | 0.03340 |
| Hydroxypropionylcarnitine | Up in CS-6mo compared to AC-6mo | 5.0x10^-4 |
|  | Up in CS-cessation compared to AC-6mo | 1.2x10^-4 |
| Linolenyl palmitate | Down in CS-6mo compared to AC-6mo | 0.000754 |
|  | Down in CS-cessation compared to AC-6mo | 0.01346 |
| TG(52:1) | Down in CS-6mo compared to CS-4mo | 0.02150 |
| TG(60:12) | Down in CS-6mo compared to CS-4mo | 0.01977 |
| TG(60:7) | Down in CS-6mo compared to AC-6mo | 0.03069 |
|  | Up in CS-cessation compared to CS-6mo | 1.7x10^-4 |

**Table 5: Differentially regulated fatty acid esters putatively identified in the plasma of the smoking mouse model**.

| **Prenol lipids** | **Regulation(s) and comparison(s)** | ***p*-value** |
| --- | --- | --- |
| 4α-formyl-4β-methyl-5α-cholesta-8-en-3β-ol | Up in CS-6mo compared to CS-4mo | 0.04016 |
| Ubiquinol 8 | Up in CS-4mo compared to AC-4mo | 0.00563 |
| β-1,4-D-Mannosylchitobiosyldiphosphodolichol | Down in CS-4mo compared to AC-4mo | 0.02825 |
| (3b,5a,6b,22a,25R)-Furostane-22-methoxy-3,6,26-triol 3-[glucosyl-(1,2)-[xylosyl-(1,3)]-glucosyl-(1,)-galactoside] 26-glucoside | Down in CS-cessation compared to AC-6mo | 0.03427 |
| 4,4-Dimethylcholesta-8,14,24-trienol | Down in CS-6mo compared to AC-6mo | 0.01224 |

**Table 6: Differentially regulated prenol lipids putatively identified in the plasma of the smoking mouse model**.
