# Supplementary material for: Transient and Persistent Metabolomic Changes in Plasma following Chronic Cigarette Smoke Exposure in a Mouse Model
Source: PLoS One. 2014 Jul 9;9(7):e101855. doi: 10.1371/journal.pone.0101855 (PMC4090193; doi:10.1371/journal.pone.0101855)
Supplement: Table S4 — Unannotated Metabolites. Metabolites which passed statistical and fold change analysis but remained unidentified based on MS library searches. (DOCX) [file pone.0101855.s005.docx]

**Supplemental Table 4**: Additional differentially regulated metabolites

Unannotated metabolites which passed FDR < 0.05 statistical significance and fold change > 1.5 analysis in the smoking comparisons in mouse plasma.

| **m/z** | **Retention Time** | **Fraction Detected** | **Comparison** | **Regulation** |
| --- | --- | --- | --- | --- |
| 81.0696 | 15.254 | Aqueous | *Air Control 4 vs. Smoking 4; Smoking 6 vs. Smoking 4* | Down; Up |
| 124.04073 | 1.023 | Aqueous | *Air Control 4 vs. Smoking 4* | Up |
| 159.09021 | 2.687 | Aqueous | *Smoking 6 vs. Smoking 4* | Up |
| 297.14102 | 5.576 | Aqueous | *Air Control 6 vs. Smoking 6; Air Control 6 vs. Stop Smoking 6* | Up; Up |
| 334.14102 | 5.903 | Aqueous | *Smoking 6 vs. Smoking 4* | Up |
| 462.26685 | 7.947 | Aqueous | *Air Control 6 vs. Stop Smoking 6* | Up |
| 475.1966 | 6.234 | Aqueous | *Air Control 6 vs. Stop Smoking 6* | Down |
| 555.0639 | 1.197 | Aqueous | *Smoking 6 vs. Smoking 4* | Down |
| 579.53265 | 7.356 | Phospholipid | *Air Control 6 vs. Stop Smoking 6* | Up |
| 599.0744 | 6.420 | Aqueous | *Smoking 6 vs. Stop Smoking 6* | Down |
| 604.31476 | 5.167 | Phospholipid | *Air Control 6 vs. Stop Smoking 6* | Down |
| 624.76465 | 6.322 | Aqueous | *Smoking 6 vs. Stop Smoking 6* | Down |
| 631.5946 | 6.308 | Aqueous | *Smoking 6 vs. Smoking 4* | Up |
| 657.02026 | 6.010 | Aqueous | *Smoking 6 vs. Smoking 4* | Up |
| 658.8316 | 5.804 | Aqueous | *Air Control 6 vs. Smoking 6* | Down |
| 690.6613 | 6.511 | Aqueous | *Smoking 6 vs. Smoking 4* | Up |
| 729.869 | 6.233 | Aqueous | *Smoking 6 vs. Smoking 4* | Up |
| 752.78937 | 6.046 | Aqueous | *Air Control 6 vs. Stop Smoking 6* | Up |
| 754.4859 | 5.694 | Phospholipid | *Smoking 6 vs. Smoking 4* | Down |
| 861.3346 | 5.720 | Aqueous | *Smoking 6 vs. Smoking 4* | Up |
| 861.3836 | 6.065 | Aqueous | *Smoking 6 vs. Smoking 4* | Up |
| 873.2856 | 15.170 | Aqueous | *Air Control 4 vs. Smoking 4* | Down |
| 878.4141 | 6.064 | Aqueous | *Smoking 6 vs. Smoking 4* | Up |
| 886.8035 | 10.681 | Neutral lipid | *Air Control 6 vs. Smoking 6* | Up |
| 915.3115 | 6.344 | Aqueous | *Smoking 6 vs. Smoking 4* | Down |
| 916.7632 | 9.914 | Neutral lipid | *Air Control 6 vs. Smoking 6* | Down |
| 949.9648 | 6.654 | Aqueous | *Smoking 6 vs. Smoking 4* | Up |
| 970.8414 | 10.918 | Neutral lipid | *Air Control 6 vs. Stop Smoking 6* | Down |
| 1085.6531 | 4.317 | Phospholipid | *Smoking 6 vs. Smoking 4* | Up |
| 1353.3721 | 10.085 | Neutral lipid | *Air Control 6 vs. Stop Smoking 6* | Up |
| 1386.0704 | 7.784 | Neutral lipid | *Air Control 4 vs. Smoking 4* | Up |
| 1508.984 | 4.512 | Phospholipid | *Smoking 6 vs. Smoking 4* | Up |
| 176.07402, 198.05957 | 1.697 | Aqueous | *Smoking 6 vs. Smoking 4* | Up |
| 257.2501, 279.22763 | 15.162 | Aqueous | *Smoking 6 vs. Smoking 4* | Up |
| 367.1488, 389.1318 | 2.793 | Aqueous | *Air Control 4 vs. Smoking 4; Smoking 6 vs. Smoking 4* | Down; Up |
| 444.279, 466.25287 | 5.904 | Aqueous | *Smoking 6 vs. Smoking 4* | Up |
| 467.73914, 489.7177 | 5.755 | Aqueous | *Air Control 4 vs. Smoking 4* | Down |
| 483.09525, 505.08438 | 1.965 | Aqueous | *Smoking 6 vs. Smoking 4* | Down |
| 507.31326, 529.2911 | 6.618 | Aqueous | *Smoking 6 vs. Smoking 4* | Up |
| 519.12476, 497.15015 | 6.453 | Aqueous | *Smoking 6 vs. Smoking 4* | Up |
| 540.3725, 562.3461 | 6.695 | Aqueous | *Smoking 6 vs. Smoking 4* | Up |
| 587.6406, 609.6254 | 6.494 | Aqueous | *Smoking 6 vs. Smoking 4* | Up |
| 597.3151, 619.2967 | 6.343 | Aqueous | *Smoking 6 vs. Smoking 4* | Up |
| 598.31055, 620.29724 | 6.307 | Aqueous | *Smoking 6 vs. Smoking 4* | Up |
| 609.84796, 631.8354 | 6.208 | Aqueous | *Smoking 6 vs. Smoking 4* | Up |
| 619.2834, 641.2709 | 6.645 | Aqueous | *Smoking 6 vs. Smoking 4* | Up |
| 633.76483, 655.7455 | 5.975 | Aqueous | *Air Control 6 vs. Stop Smoking 6* | Up |
| 635.0719, 307.04105 | 1.954 | Aqueous | *Smoking 6 vs. Smoking 4* | Down |
| 644.7629, 666.7331 | 5.980 | Aqueous | *Smoking 6 vs. Smoking 4* | Up |
| 656.8333, 678.81085 | 6.176 | Aqueous | *Smoking 6 vs. Smoking 4* | Up |
| 666.8782, 688.85815 | 6.491 | Aqueous | *Smoking 6 vs. Smoking 4* | Up |
| 690.32776, 712.3076 | 6.501 | Aqueous | *Air Control 6 vs. Smoking 6* | Up |
| 729.36676, 751.3522 | 6.231 | Aqueous | *Air Control 6 vs. Smoking 6* | Up |
| 791.3561, 813.3397 | 6.804 | Aqueous | *Smoking 6 vs. Smoking 4* | Up |
| 802.90186, 824.8866 | 6.431 | Aqueous | *Air Control 4 vs. Smoking 4* | Down |
| 810.3926, 832.3641 | 6.201 | Aqueous | *Air Control 6 vs. Smoking 6* | Up |
| 810.89874, 832.87415 | 6.228 | Aqueous | *Smoking 6 vs. Smoking 4* | Up |
| 818.57947, 841.9911 | 7.110 | Neutral | *Smoking 6 vs. Smoking 4* | Up |
| 844.4227, 866.3974 | 8.578 | Aqueous | *Smoking 6 vs. Smoking 4* | Up |
| 968.57214, 990.5444, 484.79184, 506.77112 | 6.749 | Aqueous | *Smoking 6 vs. Smoking 4* | Up |
| 575.4996 | 8.153 | Neutral | *Air Control 6 vs. Smoking 6 vs. Stop Smoking 6* | reversibly decreased with smoking |
| 577.8049, 599.7841 | 5.804 | Aqueous | *Air Control 6 vs. Smoking 6 vs. Stop Smoking 6* | reversibly decreased with smoking |
| 804.9439, 826.9301 | 6.729 | Aqueous | *Air Control 6 vs. Smoking 6 vs. Stop Smoking 6* | reversibly decreased with smoking |
| 1053.2123 | 10.252 | Neutral | *Air Control 6 vs. Smoking 6 vs. Stop Smoking 6* | reversibly decreased with smoking |
| 104.10839 | 1.066 | Neutral | *Air Control 6 vs. Smoking 6 vs. Stop Smoking 6* | reversibly increased with smoking |
| 594.8318, 616.8332 | 6.315 | Aqueous | *Air Control 6 vs. Smoking 6 vs. Stop Smoking 6* | reversibly increased with smoking |
| 729.8690 | 6.233 | Aqueous | *Air Control 6 vs. Smoking 6 vs. Stop Smoking 6* | reversibly increased with smoking |
| 891.7919 | 10.678 | Neutral | *Air Control 6 vs. Smoking 6 vs. Stop Smoking 6* | reversibly increased with smoking |
| 906.2550 | 8.719 | Neutral | *Air Control 6 vs. Smoking 6 vs. Stop Smoking 6* | reversibly increased with smoking |
| 983.2748 | 9.042 | Neutral | *Air Control 6 vs. Smoking 6 vs. Stop Smoking 6* | reversibly increased with smoking |
| 1245.8845 | 7.967 | Neutral | *Air Control 6 vs. Smoking 6 vs. Stop Smoking 6* | reversibly increased with smoking |
| 961.7057 | 10.145 | Neutral | *Air Control 6 vs. Smoking 6 vs. Stop Smoking 6* | reversibly increased with smoking |
| 646.8106 | 6.277 | Aqueous | *Air Control 6 vs. Smoking 6 vs. Stop Smoking 6* | persistently increased with smoking |
| 1353.3721 | 10.085 | Neutral | *Air Control 6 vs. Smoking 6 vs. Stop Smoking 6* | persistently increased with smoking |
| 540.3725, 562.3461 | 6.695 | Aqueous | *Air Control 6 vs. Smoking 6 vs. Stop Smoking 6* | persistently increased with smoking |
| 826.45294 | 9.994 | Neutral | *Air Control 6 vs. Smoking 6 vs. Stop Smoking 6* | persistently decreased with smoking |
| 888.09686 | 9.898 | Neutral | *Air Control 6 vs. Smoking 6 vs. Stop Smoking 6* | persistently decreased with smoking |
| 1206.8606 | 7.891 | Neutral | *Air Control 6 vs. Smoking 6 vs. Stop Smoking 6* | persistently decreased with smoking |

Multiple m/z in column indicates additional adducts with comparable intensity levels for the unidentified compound
